# Mechanisms of *in vivo* binding site selection of the hematopoietic master transcription factor PU.1

**DOI:** 10.1093/nar/gkt355

**Published:** 2013-05-08

**Authors:** Thu-Hang Pham, Julia Minderjahn, Christian Schmidl, Helen Hoffmeister, Sandra Schmidhofer, Wei Chen, Gernot Längst, Christopher Benner, Michael Rehli

**Affiliations:** ^1^Department of Internal Medicine III, University Hospital Regensburg, F.-J.-Strauss Allee 11, D-93042 Regensburg, Germany, ^2^Department of Biochemistry III, University of Regensburg, Universitätsstrasse 31, D-93053 Regensburg, Germany, ^3^Berlin Institute for Medical Systems Biology (BIMSB), Max Delbrück Center for Molecular Medicine (MDC) Berlin-Buch, D-13092 Berlin, Germany, ^4^Department of Cellular and Molecular Medicine, University of California San Diego, La Jolla, CA 92093-0651, USA and ^5^Integrative Genomics and Bioinformatics Core, Salk Institute for Biological Studies, La Jolla, CA 92037, USA

## Abstract

The transcription factor PU.1 is crucial for the development of many hematopoietic lineages and its binding patterns significantly change during differentiation processes. However, the ‘rules’ for binding or not-binding of potential binding sites are only partially understood. To unveil basic characteristics of PU.1 binding site selection in different cell types, we studied the binding properties of PU.1 during human macrophage differentiation. Using *in vivo* and *in vitro* binding assays, as well as computational prediction, we show that PU.1 selects its binding sites primarily based on sequence affinity, which results in the frequent autonomous binding of high affinity sites in DNase I inaccessible regions (25–45% of all occupied sites). Increasing PU.1 concentrations and the availability of cooperative transcription factor interactions during lineage differentiation both decrease affinity thresholds for *in vivo* binding and fine-tune cell type-specific PU.1 binding, which seems to be largely independent of DNA methylation. Occupied sites were predominantly detected in active chromatin domains, which are characterized by higher densities of PU.1 recognition sites and neighboring motifs for cooperative transcription factors. Our study supports a model of PU.1 binding control that involves motif-binding affinity, PU.1 concentration, cooperativeness with neighboring transcription factor sites and chromatin domain accessibility, which likely applies to all PU.1 expressing cells.

## INTRODUCTION

Transcription factors are defined through their ability to recognize and bind specific sequence motifs in genomic DNA. In a nuclear environment, the access of transcription factors to potential binding sites is highly restricted and only a relatively small proportion of regulatory elements are effectively bound. This selectivity has largely been attributed to restrictive features of chromatin (both on nucleosome- or higher-order structure levels) or DNA methylation. The ability to overcome chromatin restriction, at least on nucleosome level, may be a key feature of master regulators (also called ‘pioneering factors’), whereas a second tier of transcription factors is thought to primarily gain access to binding sites that are already ‘made accessible’ by master regulators. This view is supported by several recent studies reporting the genome-wide and cell type-specific co-localization of transcription factors (1–10). Specific features of master regulators that would enable them to overcome chromatin restriction remain to be defined but may include their capability to engage co-factors like chromatin remodeling complexes, as well as epigenetic modifiers to create poised or activated chromatin states that are then accessible to other factors. Alternatively (or additionally), they may compete more efficiently with nucleosomes for DNA binding than other factors, either autonomously or in cooperation with other factors.

A well-studied example of such a master regulator is the transcription factor PU.1. This factor is restricted to the hematopoietic compartment and is required for the generation of common lymphoid and granulocyte–macrophage progenitor cells, as well as later stages of monocyte/macrophage and B-cell development (11). Recent studies have reported the cell type-specific distribution of PU.1-binding sites in mouse lymphoid and myeloid progenitor cells (12,13), mouse macrophages or B cells (8,14), murine reticulocytes (15,16), as well as in human monocytes and macrophages (7). In monocytes/macrophages and B cells, cell type-specific PU.1 binding is characterized by the co-occurrence of sequence motifs for additional cell type-specific transcription factors. For example, in murine macrophages, PU.1 frequently associates with the nearby binding of transcription factors C/EBPα/β and AP-1, whereas in murine B cells, PU.1 binding occurs in combination with a distinct set of B-cell-specific factors, including E2A, EBF and OCT2 (8). Additional evidence suggests that signaling-associated factors like NF-κB, SMAD, PPARγ or the LXR transcription factors preferentially access regions pre-bound by the master regulator PU.1, which may explain at least some of the cell type-specific signaling responses observed in different PU.1 expressing cell types (2,4,8,14). In analogy to the aforementioned studies, we recently defined a human macrophage-specific enhancer signature, which included PU.1, CEBP, bZIP, EGR, E-Box and NF-κB motifs and showed that macrophage-specific PU.1 binding in humans occurs in combination with the corresponding factors binding the aforementioned motifs (7).

In addition to cell type-specific binding sites, which are mainly found at promoter distal sites, there is a core set of PU.1 occupied sites that is shared between hematopoietic cell types. These sites are frequently associated with motifs for promoter-located general transcription factors and likely regulate genes that are commonly expressed (or repressed) in hematopoietic lineages. However, only a fraction of common and cell type-specific binding events of the master regulator PU.1 can be explained by cooperativeness between neighboring transcription factor binding sites. In addition, it is also unclear why only a strikingly small fraction of potential binding sites across the entire genome is occupied by PU.1. In general, the prerequisites for DNA binding of master regulators like PU.1 are insufficiently understood.

By studying the properties of PU.1 recognition sites *in vitro*, as well as the distribution of PU.1-bound sites during monocyte differentiation across the genome *in vivo*, we unveil three major classes of recognition sites that are defined by motif affinity, cooperativeness, PU.1 concentration and higher-order chromatin structure. DNA methylation seems to play a minor role in restricting DNA binding of the master regulator PU.1 (at least in accessible chromatin domains) and instead is locally erased in the vicinity of transcription factor-bound sites. The fundamental dependency of PU.1 on motif-binding affinity distinguishes this pioneering factor from many other transcription factors that are mainly guided by open chromatin and bind in combination with other transcription factors. These findings not only explain the large majority of PU.1-binding events during monocyte differentiation but will also be relevant to other PU.1 expressing cell types.

## MATERIALS AND METHODS

### Ethics statement

Collection of blood cells from healthy donors was performed in compliance with the Helsinki Declaration. All donors signed an informed consent. The leukapheresis procedure and subsequent purification of hematopoietic cell types were approved by the local ethical committee (reference number 92–1782 and 09/066c).

### Cells

Separation of peripheral blood cell types and *in vitro* differentiation of monocytes into macrophages were done as described previously (7,17).

### Chromatin immunoprecipitation

Chromatin immunoprecipitation (ChIP) experiments were carried out as described previously (7). The anti-CTCF antibodies used for ChIP were kindly provided by Victor Lobanenkov (NIAID).

### DNA methylation analyses

Methyl-CpG immunoprecipitation (MCIp) was performed as described previously (18,19) using 2 µg of genomic DNA as starting material. Densely CpG-methylated DNA fragments were recovered from MBD-Fc-beads for high-throughput sequencing after washing with 550 mM NaCl. Mass spectrometry (MS) analysis of bisulfite-converted DNA was done as described previously (18,19). Detailed descriptions and oligo sequences are provided in the Supplementary Methods.

### High-throughput sequencing and mapping

DNA from chromatin immunoprecipitation (10–50 ng) or MCIp enrichment was adapter ligated and polymerase chain reaction amplified according to the manufacturer’s protocol (Illumina, San Diego, USA). ChIP fragments were sequenced for 36 cycles on Illumina Genome Analyzers I or II according to the manufacturer’s instructions. Sequence tags of own or published experiments were mapped to the current human reference sequence (GRCh37/hg19) using Bowtie (20), and only uniquely mapped tags were used for downstream analyses. Tag counts were normalized to 10^7^ specifically mapped tags. Sequencing data have been deposited with the NCBI GEO database and accession code GSE43098. A complete list of all sequencing data sets (including accession nos.) generated and/or analyzed in this study is provided in the Supplementary Methods.

### Data analysis

Analysis of mapped ChIP-seq tags was performed using HOMER (8). ChIP-Seq quality control, transcription factor peak finding, transcription start sites (TSS) annotation (based on GENCODE V13) and motif analysis were done essentially as described previously (7,8). A detailed description of computational analyses is provided in the Supplementary Methods.

### Motif affinity measurements

Motif affinity measurements were carried out essentially as described previously (21). In brief, binding assays were performed using annealed oligonucleotides (Cy3-labeled on one strand) and recombinant full-length PU.1 on the Nanotemper Monolith NT.115 device. For each motif, two independent sets of 16 affinity measurement reactions were prepared using a dilution series of PU.1 protein and keeping the concentration of the double-stranded oligonucleotide constant. Data analysis was done using the NT-analysis acquisition software (1.2.229). A detailed description is provided in the Supplementary Methods.

## RESULTS

### PU.1 occupies a relatively small proportion of its consensus sites in progenitor cells, monocytes and macrophages

**Figure 1.**
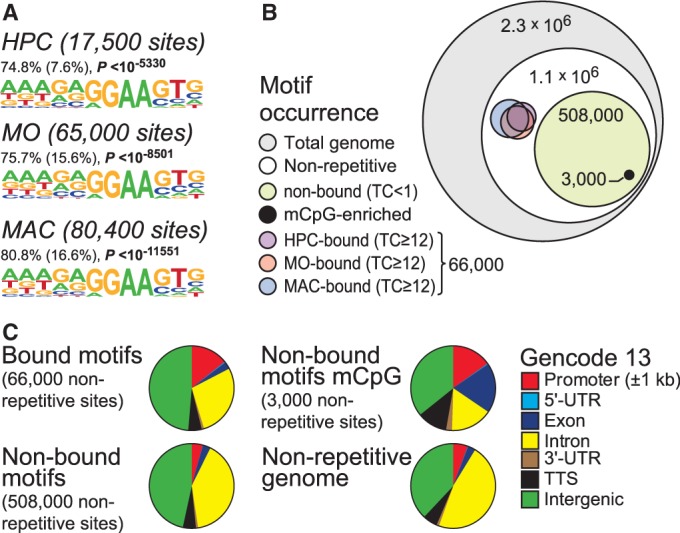
Global distribution of the PU.1 consensus motif. (**A**) *De novo* identified sequence motifs associated with PU.1 peak regions. The fraction of peak regions (200 bp) containing at least one motif instance, the expected frequency of the motif in random sequences (in parentheses), as well as *P*-values (hypergeometric) for the overrepresentation of each motif, are given. (**B**) Diagram showing the global occurrence of PU.1 consensus sites relative to the 2.1 million PU.1 consensus sites (MAC-derived motif) within the human genome (numbers indicate the sizes of motif fractions). TC, normalized tag count; mCpG-enriched, PU.1 sites that overlap with DNA methylation as detected by MCIp. (**C**) Pie charts showing the genomic distribution of the MAC-derived PU.1 consensus sites conditional on their binding status (bound or non-bound), as well as their DNA methylation status as detected by MCIp (non-bound motifs mCpG). A reference pie chart shows the overall proportion of different genomic elements in the non-repetitive human genome sequence. Annotation is based on GENCODE V13.

In primary human monocytes (MO) and macrophages (MAC), the transcription factor PU.1 shows a significant proportion of cell stage-specific–binding events despite comparable PU.1 expression levels in both cell types (7). A similar analysis using recently published data for PU.1 occupancy in CD133 positive hematopoietic progenitor cells (HPC) also revealed considerable binding dynamics between HPC and monocytes (Supplementary Figure S1). Cell types shared almost identical sequence motifs at PU.1-binding sites (Figure 1A) similar to those derived from earlier ChIP-seq studies (8,16,22). The human macrophage-derived consensus sequence was most comprehensive and covered up to 80% of all PU.1 ChIP-seq peaks identified in HPC, MO or MAC, corresponding to 66 000 genomic locations. To compare characteristics of bound and non-bound regions, we mapped the MAC-derived PU.1 consensus motif throughout the genome and identified 2.1 × 10^6^ possible recognition sites, with 1.1 × 10^6^ sites located in non-repetitive regions, suggesting that only a relatively small proportion of possible binding sites (6% of the non-repetitive genome) is actually bound by PU.1 during macrophage differentiation (Figure 1B). Roughly 0.5 × 10^6^ sites showed absolutely no sign of binding [Chip-seq tag count (TC) <1 in a 200-bp motif-centered window], which we considered the ‘non-bound’ fraction of the human genome. Given that there is no obvious difference in the recognition sites and the fact that only a fraction of recognition sites is actually bound, one may raise the question why certain sites are only bound in one cell type or not at all. To address these issues, we systematically compared features of bound and non-bound recognition sites.

### General characteristics of bound and non-bound PU.1 consensus sites

**Figure 2.**
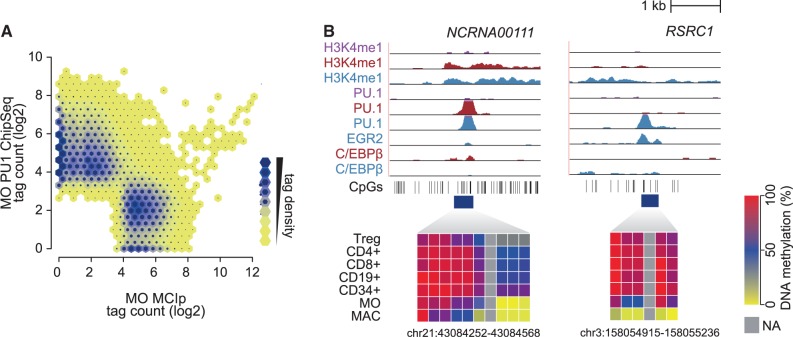
Relationship between transcription factor binding and DNA methylation. (**A**) Monocyte MCIp-Seq tag counts (representing DNA methylation levels) are compared with corresponding PU.1 ChIP-Seq tag counts at merged peak sets in a hexbin density plot. The colors represent the relative density of peaks in each location within the density plot. (**B**) Examples for transcription factor-bound promoter-distal regions that showed induced cell stage-specific transcription factor binding were subjected to DNA methylation analysis. Indicated ChIP-Seq tracks for HPC (purple), monocytes (red) and macrophages (blue) are shown for each region (top). Positions of CpG dinucleotides are indicated as vertical lines below the tracks, and regions analyzed by Matrix-assisted laser desorption/ionization (MALDI)-time-of-flight (TOF)–mass spectrometrie (MS) of bisulfite-converted DNA from the indicated blood cell types are indicated by the dark blue boxes. Heat maps depict the methylation status of individual CpGs from red (100%) over blue (50%) to yellow (0%), with each box representing a single CpG. Data of at least three independent donors were averaged.

We initially compared genomic locations, as well as epigenetic and sequence features for bound and non-bound consensus motifs. Genome ontology analyses (Figure 1C and Supplementary Figure S2) suggested that PU.1-bound motifs (compared with non-bound ones) were significantly enriched at promoters and 5′ untranslated regions and most significantly depleted in gene deserts (Supplementary Figure S2). In line with previous observations (7), PU.1-bound motifs in HPC, MO or MAC showed significant local enrichment of DNase I cleavage sites, as well as active histone marks, including H3K4me1-3, H3K27ac, H3K9ac and H2AZ, whereas the repressive H3K27me3 mark was locally depleted (Supplementary Figure S3). As DNA methylation could be one of the factors restricting PU.1 binding, we analyzed DNA methylation in human monocytes using methyl-CpG-immunoprecipitation coupled to next-generation sequencing (MCIp-seq), an assay that applies recombinant MBD2-Fc for the enrichment of methylated DNA (19). Even the large fraction of non-bound motifs only showed a small overlap (<1%) with MCIp-enriched DNA-methylated regions (Figure 1C). Although non-bound motifs were generally associated with intergenic and intronic sequences, non-bound and simultaneously DNA-methylated regions significantly correlated with transcribed units (exons, introns and 3′-untranslated regions (UTR)), suggesting that DNA methylation in gene bodies may prevent internal transcription factor binding, e.g. to avoid the generation of alternative TSS (Figure 1C). The limited overlap of methylated regions with transcription factor-bound regions in monocytes (Figure 2A and Supplementary Figure S4A) concurs with the preferentially low CpG content surrounding PU.1-binding sites (Supplementary Figure S4B). Although the mutual exclusiveness of DNA methylation and PU.1 binding does not distinguish cause and consequence, we also observed DNA demethylation at sites that acquired PU.1 binding in MO or MAC (Figure 2B and additional examples in Supplementary Figure S4C and D).

DNA demethylation events during post-mitotic monocyte to macrophage differentiation are active (23), suggesting that DNA methylation is actively removed (at least in ‘accessible’ low to intermediate CpG density regions) and does not necessarily impede DNA binding of PU.1. The observed demethylation is spatially limited and, therefore, likely initiated by sequence-dependent transcription factor recruitment, which is in general agreement with recently published comparisons of transcription factor binding and DNA methylation (24,25).

We also studied the composition of sequence motifs around bound and non-bound PU.1 consensus sites. In line with previous work (7,8), the collection of PU.1-bound motifs from each cell stage was significantly associated with consensus sequences for ubiquitous (CTCF, SP1 and NRF1), as well as cell stage-specific factors (like RUNX1, AP1, CEBP-family factors and so forth) (Supplementary Figure S5). Bound non-consensus sites were overrepresented at CpG islands (CGI) and showed enrichment for ETS-like motifs [which are not covered by the consensus position weight matrix (PWM) but may still be recognized by PU.1], as well as an even higher enrichment of the same co-associated non-canonical motifs that are also observed at consensus sites (Supplementary Figure S5), indicating that the detected PU.1-binding events are rarely tethered. Non-bound PU.1 recognition sites were mainly enriched for AT-rich sequence motifs resembling consensus sites of homeotic transcription factors that are involved in developmental processes (Supplementary Figure S5), as well as an E-box site resembling the E2A sequence observed in the vicinity of B cell-specific PU.1-binding sites (8). This indicated that non-bound motifs in HPC, MO and MAC are frequently located in genome areas (or chromatin domains) containing genes that are likely developmentally silenced or only activated in other PU.1 expressing cells like B cells for example.

**Figure 3.**
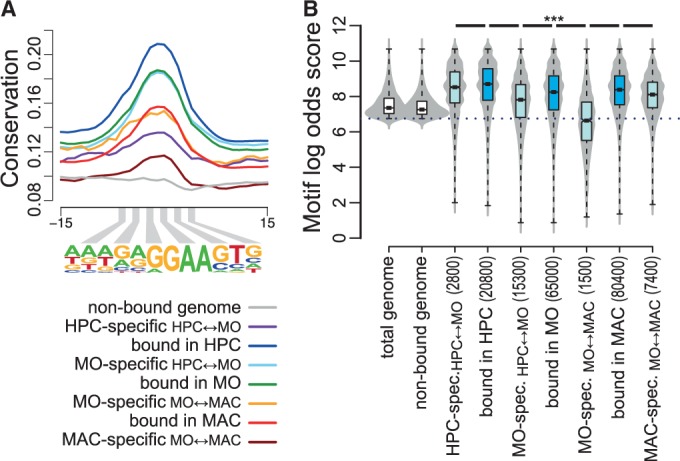
Motif conservation and motif score classes of bound and non-bound PU.1 consensus sites. (**A**) Histogram of average per-nucleotide vertebrate conservation (PhastCons) surrounding non-bound motifs, HPC-specific motifs (relative to MO), all HPC-bound motifs, MO-specific motifs (relative to HPC), all MO-bound motifs, MO-specific motifs (relative to MAC), all MAC-bound motifs and MAC-specific motifs (relative to MO), as indicated by coloring. (**B**) Combined bean and box plot showing the distribution of motif log-odds scores of annotated PU.1 motifs (white boxes) or best scoring motifs within total (blue boxes) or cell type-specific PU.1 peaks (light blue boxes). Solid bars of boxes display the interquartile ranges (25–75%) with an intersection as the median; whiskers represent max/min values. Significantly different motif score distributions in pairwise comparisons are indicated (***P < 0.001, Mann–Whitney U-test, two-sided). The detection threshold is indicated by the dotted line.

We next focused on PU.1-binding sites that are dynamically bound during monocyte differentiation and studied the sequence conservation across vertebrates, as well as the distributions of motif log-odds scores (representing the degree of deviation from the consensus motif) of non-bound and bound PU.1 motifs. Bound motifs were separated into cell type-specific motifs (HPC-specific relative to MO, MO-specific relative to HPC or MAC, as well as MAC-specific relative to MO), or all motifs bound in a given cell type. As shown in Figure 3A, vertebrate conservation was generally higher for all PU.1 peaks in a given cell type, with HPC-bound peaks showing the highest degree of conservation and MAC- and HPC-specific–binding sites showing the lowest degree of conservation.

The global analysis of all mapped occurrences of the PU.1 consensus sequence across the genome revealed that motif scores were generally low at non-bound sites (Figure 3B). In contrast, bound sequences in either cell type shared intermediate to high motif scores, with HPC (expressing the lowest levels of PU.1) showing the highest log-odd scores in bound elements. Motif scores of differentially bound sites were significantly lower than those of total bound sites (Figure 3B). Lowest motif scores in MO-specific peaks (relative to MAC) reflect their high content of binding sites in CpG islands that are frequently not covered by the consensus PWM. These observations were independent of the motif source, as alternative PU.1 PWM essentially showed the same motif score distribution across samples (Supplementary Figure S6). As we know that cell stage-specific PU.1 recruitment requires stabilization through transcription factor cooperativeness, the observed distributions suggested a possible correlation between motif scores and motif affinity.

### Influence of motif affinity on PU.1 binding *in vitro* and *in vivo*

**Figure 4.**
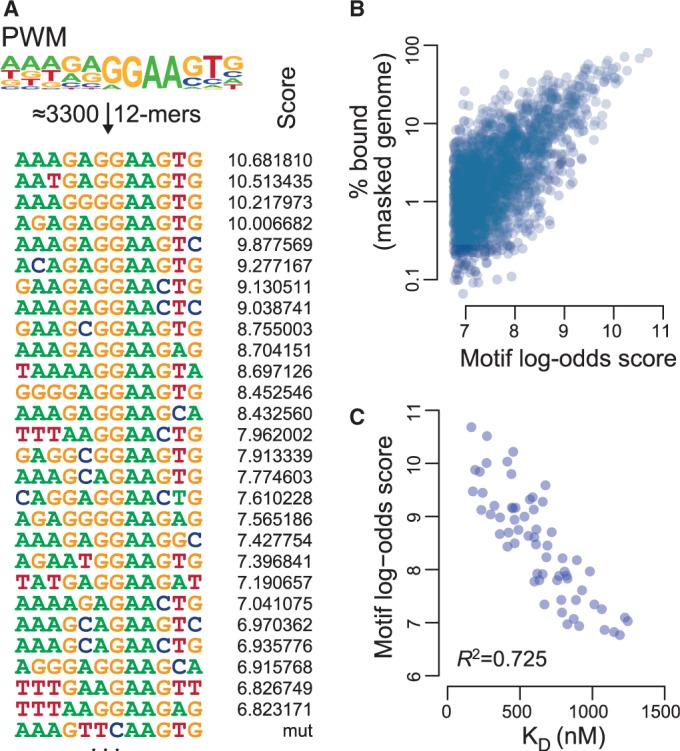
Motif scores correlate with *in vitro* binding affinity. (**A**) The ChIP-seq–derived PU.1 consensus PWM (sequence logo is shown on top) comprises ∼3300 different 12mers. Twenty-five representative 12mers overlapping the PWM are shown below the logo next to their motif log-odds scores. (**B**) Scatter plot showing scores of motifs relative to the frequency of *in vivo* binding events across the repeat-masked genome (only motifs with >100 occurrences across the masked genome are included). (**C**) Scatter plot showing the correlation between microscale thermophoresis-derived dissociation constants (*K*_D_-values) for the interaction between recombinant full-length PU.1 and double-stranded oligonucleotides [including the ones listed in (A)] with corresponding motif log-odds scores. Only *K*_D_-values <1500 mM were plotted. Oligos with larger *K*_D_-values (between 1500 and 4500 mM, 12 in total) had low-log-odds scores (between 6.7 and 7.6). A complete list of *K*_D_- and log-odds score values is given in Supplementary Table S1.

As the affinity between a transcription factor and its recognition sequence is a proximate determinant of DNA binding, we further studied its relationship with motif scores. The PU.1 consensus PWM comprises ∼3300 individual 12mers (some examples are given in Figure 4A) with highly variable rates of *in vivo* occupancy. Although *in vivo* binding is naturally influenced by DNA–transcription factor interactions, it still roughly correlated with motif log-odds scores (Figure 4B). This type of correlation has previously also been made in other systems (26,27), substantiating the hypothesis that PU.1 motif scores would reflect motif affinity. To address this further, we initially compared published binding data for the PU.1 DNA-binding domain with 8mer sequences on universal protein binding microarrays (28) and observed a good correlation (*R*^2 ^= 0.59) between microarray signal intensity *Z*-scores and max log-odds scores for overlapping 12mers (Supplementary Figure S7). However, as the motif PWM is longer than the sequences used in the microarray study (12 versus 8 bp), we experimentally assayed the relationship between log-odds scores of selected 12mers and their affinity to full-length PU.1 in solution. In total, we determined dissociation constants (*K*_D_ values) for 75 individual sequence motifs and bacterially expressed full-length PU.1 in solution using microscale thermophoresis. This assay detects interaction-dependent changes in the hydration shell of molecules, as well as their changes in thermophoretic mobility in solution. The differential diffusion of the substrate or the substrate with its bound interaction partner can be quantified via fluorescent labels (21,29). As shown in Figure 4C, log-odds scores of individual motifs show an inverse correlation with *in vitro K*_D_ values (coefficient of determination *R*^2 ^= 0.725, results of all measurements are given in Supplementary Table S1), suggesting that the motif log-odds scores indeed represent a good measure for motif affinity. Our data also suggest that all 12 positions of the PWM can affect motif affinity.

### Cooperativeness with neighboring sites and DNase I accessibility

**Figure 5.**
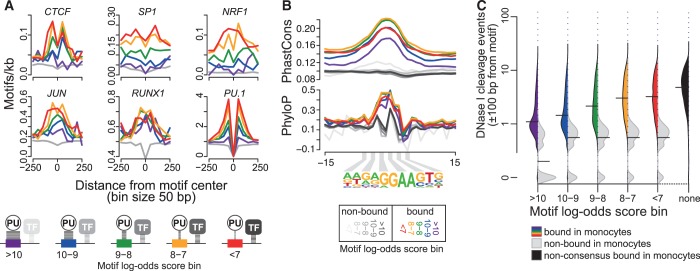
Motif co-association, evolutionary conservation and chromatin accessibility in relation to motif affinity. (**A**) Histograms showing the distribution of indicated consensus motifs around PU.1 motifs as a function of motif scores. Motif score classes are indicated by coloring, the gray lines represent motif distributions around non-bound sites. The schematic below illustrates the inverse relationship between motif scores (affinity) and cooperativeness. (**B**) Histograms showing average per-nucleotide vertebrate conservation (PhastCons and PhyloP) surrounding motifs belonging to different score classes. (**C**) Bean plots showing the distribution of DNase I cleavage frequency around PU.1 bound (consensus site: colored filling, non-consensus sites: black filling) and non-bound motifs (gray filling) depending on motif score classes. DNase I cleavage events (at nucleotide resolution, tag counts normalized to 10^7^) were counted in a 200-bp window around each motif. Horizontal bars mark the median of each distribution. DNase I cleavage data (representing four independent donors) were originally generated by the ENCODE or the Roadmap Epigenomics projects (for accession nos. see the Supplementary Methods). A corresponding plot for HPC is shown in Supplementary Figure S9A.

We next compared motif affinity ranges (represented by log-odds scores) with their levels of motif co-association, evolutionary conservation and local chromatin accessibility (represented by DNase I cleavage sensitivity). As shown in Figure 5A, the top co-associated motifs were most frequent around intermediate/low affinity motifs, suggesting that the requirement for cooperativeness increases with decreasing motif affinity. The inverse correlation between affinity and levels of co-association was most striking at CpG-rich motifs (like CTCF, SP1 and NRF1). As noted previously (15,30), PU.1 motifs also frequently appear in pairs or even clusters of three or more motifs. PU.1 motif clusters are bound more often, are more conserved and show higher PU.1 tag counts than single motifs (Supplementary Figure S8) implying functional importance. PU.1-bound motif clusters were generally enriched for ETS motifs and depleted for other top co-associated motifs, suggesting that PU.1 itself or other ETS factors may also act as cooperative partners (Supplementary Figure S8). Motif pairs were more likely bound if they contained at least one high or intermediate score motif, whereas combinations of low score motifs were rarely bound (Supplementary Figure S8), suggesting similar affinity requirements for cooperative binding at homotypic and heterotypic motif clusters.

PhyloP and PhastCons conservation scores were highest in the intermediate score ranges (score between 7 and 9), whereas motifs with highest affinities (score >10) showed the lowest degree of regional conservation (Figure 5B). As shown in Figure 5C, we also observed an inverse relationship between DNA accessibility and motif affinity, demonstrating that low-affinity (low score) motifs more frequently reside in highly accessible chromatin, whereas high-affinity motifs may also bind genomic regions that are less accessible. Interestingly, bound non-canonical binding sites (PU.1 peaks not covered by the consensus PWM and likely representing very low-affinity sites) showed the highest average accessibility. As a high degree of accessibility indicates the presence of additional factors, this further implies that low-affinity PU.1 motifs require cooperativeness for binding.

By comparing DNase I cleavage patterns [publicly available for MO and HPC, (31)] with PU.1 occupancy, we noticed a substantial proportion of PU.1-binding sites that showed no overlap with DNase I cleaved regions. To simplify matters, we call the latter category of binding sites DNase I non-accessible. However, it should be noted that the DNase-seq protocol applied to MO and HPC samples, which is based on the isolation of DNase I double-hit fragments (32), may underestimate the number of small accessible regions. It should also be noted that the PU.1 binding and DNase I accessibility data were from overlapping but not identical HPC populations (CD133+ and CD34+ progenitors, respectively), which may limit their comparability.

**Figure 6.**
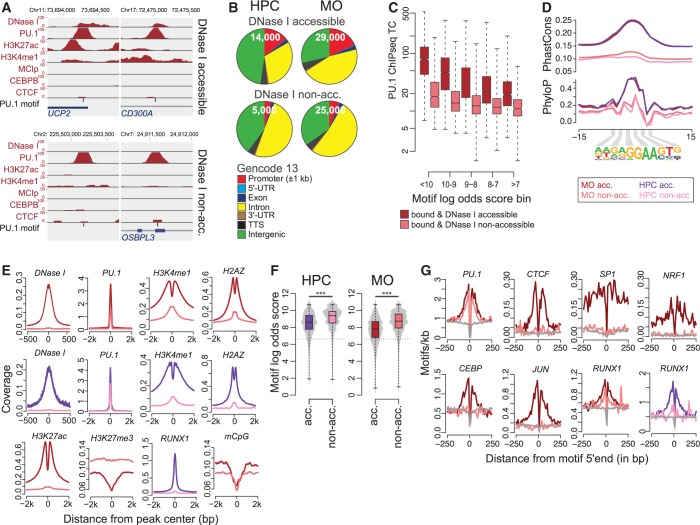
Features of DNase I accessible or non-accessible bound PU.1 consensus sites. (**A**) UCSC genome browser tracks showing examples for DNase I accessible (top) and non-accessible high-affinity PU.1-binding sites (bottom). (**B**) Pie charts showing the genomic distribution of PU.1 consensus motifs conditional on their DNase I accessibility. Annotation is based on GENCODE V13. (**C**) ChIP-seq tag counts in MO PU.1 peaks conditional on DNase I accessibility and motif score classes. (**D**) Histograms for genomic distance distributions of the indicated sequencing data sets centered across accessible or non-accessible PU.1 consensus sites across a 4-kb (or 1-kb) genomic interval. Cell types and accessibility states are indicated by coloring as shown in (**E**). (E) Histograms showing average per-nucleotide vertebrate conservation (PhastCons and PhyloP) surrounding accessible or non-accessible motifs in HPC or MO. (**F**) Combined bean and box plot showing the distribution of motif log-odds scores of accessible or non-accessible PU.1 consensus sites in HPC and MO. Solid bars of boxes display the interquartile ranges (25–75%) with an intersection as the median; whiskers represent max/min values. Motif score distributions in pairwise comparisons are highly significant (***P < 0.001, Mann–Whitney U-test, two-sided). The detection threshold is indicated by the dotted line. (**G**) Histograms showing the distribution of indicated consensus motifs around bound accessible or non-accessible PU.1 motifs, as well as non-bound motifs. Cell types and accessibility states are indicated by coloring as shown in (E); the gray lines represent motif distributions around non-bound sites.

Examples for accessible and non-accessible sites are shown in Figure 6A. DNase I accessible sites showed a significantly stronger association with promoters (Figure 6B, *P* < 10^−^^98^, hypergeometric test), were generally associated with higher PU.1 ChIP-seq tag counts (which correlated with motif affinity in both cases Figure 6C), were generally conserved (Figure 6D) and were most strongly enriched for active histone marks (Figure 6E). The proportion of DNase I non-accessible sites increased with PU.1 expression levels (HPC < MO, Figure 6B). Inaccessible sites were generally characterized by low or absent histone marking; however, both accessible and non-accessible sites were depleted for DNA methylation (Figure 6E). Strikingly, DNase I non-accessible sites showed extremely low levels of sequence conservation (Figure 6D), significantly higher motif scores (Figure 6F) and limited enrichment of co-associated motifs (Figure 6G). The synopsis of the aforementioned data strongly suggests that DNase I non-accessible sites represent autonomous PU.1-binding events that are spontaneous and almost solely driven by sequence availability and motif affinity. Our results also imply that the requirement for the presence of co-associated transcription factor binding sites for DNA binding of PU.1 increases with decreasing motif affinity. It is likely that the actual cooperativeness between motif corresponding factors is even higher in reality, as we only covered a small proportion of the expressed and possibly co-binding transcription factors, and the annotation of position weight matrices rarely covers all binding events. Interestingly, a considerable fraction of DNase I non-accessible sites (12%) becomes accessible and ‘active’ during monocyte differentiation and is more conserved than the average of autonomous sites (Supplementary Figure S9). Although it is possible that some of these sites are selected because of the integration of data from two non-identical and heterogeneous HPC populations, this observation may indicate that the ability to autonomously bind some of its recognition sites may be a key property of the master regulator PU.1.

### Motif composition in active versus inactive chromatin domains

**Figure 7.**
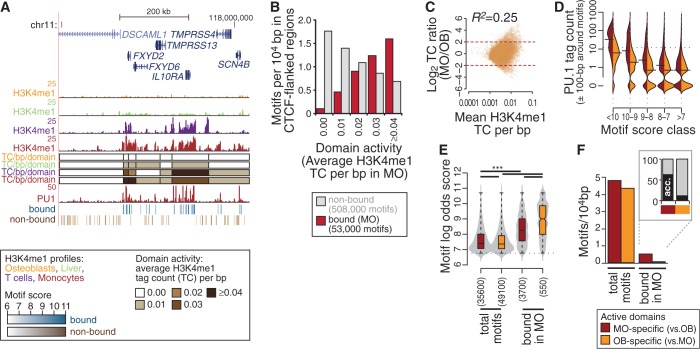
Differential distribution of bound and non-bound PU.1 consensus sites on the level of chromatin domains. (**A**) Genome browser tracks of a representative 750-kb genomic interval on chromosome 11 for H3K4me1 (osteoblasts, liver, T cells and MO) and PU.1 (MO). CTCF-flanked regions are indicated by colored boxes [coloring indicates the H3K4me1 tag density (TC/bp; normalized tag count per base pair)]. Positions of bound (blue) and non-bound (brown) consensus motifs are also provided. Motif scores in the respective tracks are indicated by color ranges (light to dark coloring corresponds to low to high scores). (**B**) Bar chart of bound (red) and non-bound (gray) motif frequencies across domain activity bins. (**C**) Tag count per base pair ratios for MO versus osteoblasts (OB) are plotted against average tag counts for CTCF-flanked domains (MvA plot). The correlation coefficient for the direct comparison of log-transformed tag counts (TC) per base pair are given above the diagram. (**D**) Distribution of normalized PU.1 ChIP-seq tag counts around motifs contingent on motif score classes in domains showing cell type-specific activity. The horizontal bar indicates the median of each distribution. The dotted line indicates the tag threshold for peaks considered bound. (**E**) Combined bean and box plot showing the distribution of motif log-odds scores for the all PU.1 motifs (total) or MO-bound PU.1 motifs within cell type-specific domains. Solid bars of boxes display the interquartile ranges (25–75%) with an intersection as the median; whiskers represent min/max. Coloring indicates the type of domain. Significantly different motif score distributions in pairwise comparisons are indicated (***P < 0.001, Mann–Whitney U test, two-sided). (**F**) Bar chart of non-bound and bound motif frequencies in OB- or MO-specific domains. The additional boxed chart shows frequencies of bound motifs overlapping DNase I accessible sites in MO.

Genome ontology analyses (Supplementary Figure S2) suggested that gene deserts were particularly rich in non-bound motifs, suggesting that opportunity of PU.1 to access individual binding sites may to some degree depend on higher-order chromatin structures. Bound sites in gene deserts comprised predominantly high-affinity sites showing little conservation and were primarily observed at non-accessible sites, suggesting that the large majority of binding events in gene deserts are autonomous and motif affinity driven, although we still observed a number of high-score motifs, which were not bound (Supplementary Figure S10). To study the distribution and features of PU.1 motifs across chromatin domains in general, we segregated the genome into intervals flanked by CTCF, a major constituent of boundary elements (33). We generated genome-wide CTCF-binding maps for MO and MAC using ChIP-seq, collected large (>10 000 bp) CTCF-flanked regions and used the mean H3K4me1 tag count across domains as a surrogate measure for domain ‘activity’. An example for this type of domain segregation is shown in Figure 7A for MO and three non-PU.1 expressing cell types.

Domain activity generally correlated with gene expression levels of domain-associated genes (Supplementary Figure S11), and the frequency of PU.1-binding events in MO (Figure 7B) increased with domain activity (similar analyses for MAC and T cells in Supplementary Figure S12) across the range of motif affinity classes (Supplementary Figure S13). A similar correlation was observed for domain activities in non-PU.1 expressing T cells (Supplementary Figure S12B), indicating that the domain activity is at least partially independent of PU.1. As observed for gene deserts, PU.1 binding in domains with low or no H3K4me1 deposition was mainly observed at intermediate/high-score motifs that are frequently affinity driven and not accessible to DNase I (Supplementary Figure S14). Similar observations were made for domains with cell type-specific ‘activity’ states. Between the closely related cell types HPC, MO and MAC, the H3K4me1 tag count distribution over chromatin domains was similar, and few domains with a large (≥4-fold) activity differences were identified (Supplementary Figure S15). Domain activity differences were much stronger between unrelated cell types as shown for osteoblasts (34) in Figure 7C or liver (data from the Roadmap epigenomics project) in Supplementary Figure S16, revealing several hundred domains showing large (≥4-fold) cell type-specific differences in H3K4me1. Notably, the PU.1 TC and motif score distributions (Figure 7D and E; liver data in Supplementary Figure S16B and C) were strikingly different in chromatin domains showing cell type-specific activity. The few sites within osteoblast- or liver-specific domains that were bound by PU.1 in monocytes had a high average motif score and were mainly DNase I non-accessible (Figure 7E and F and Supplementary Figure S16C and D), resembling the motif features in inactive domains and gene deserts.

**Figure 8.**
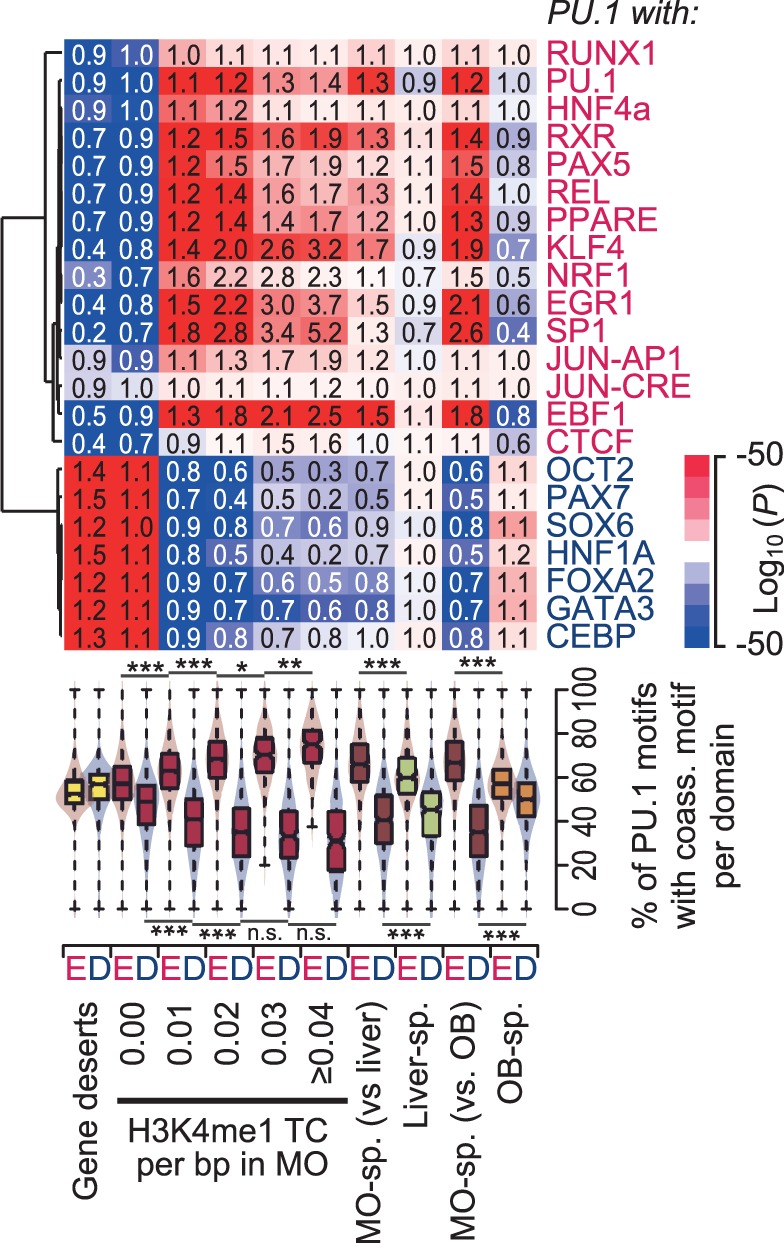
Differential distribution of sequence motif combinations on the level of chromatin domains. The top shows the hierarchical clustering (Pearson correlation uncentered, average linkage) of significance values for motif co-enrichment of the indicated consensus motifs and the PU.1 consensus motif in the indicated domain categories. *P*-values for motif co-enrichment were calculated using the hypergeometric test relative to the distribution in the total repeat-masked set. Characteristics of individual domain categories are summarized in Supplementary Table S2. Data are presented as a heatmap where blue (red) coloring indicates a significant enrichment (depletion) of motif co-occurrence. Numbers in boxes represent corresponding relative changes in motif co-enrichment. The combined bean and box plot below indicate the frequency distribution of PU.1 motifs that are associated with at least one of the enriched (red, E) or depleted (blue, D) motifs within a 100-bp window, relative to all PU.1 motifs in a domain.

The distribution of motifs and their scores generally seemed uneven across domains in different categories, implying that active domains may be characterized by a specific motif composition. We, therefore, calculated both the PU.1 motif densities and the frequencies of co-associated motifs across domains. As shown in the heatmap of Figure 8, active domains were clearly enriched for a set of co-associated transcription factor motifs (E-motifs) relevant for PU.1 expressing cells (like REL, KLF4, JUN, PPAR and so forth), whereas other motifs (including OCT, PAX7, SOX6, FOXA2 and so forth) were clearly depleted (D-motifs). Inactive domains or domains active in other cell types mostly showed an inverse enrichment pattern. The distribution plots shown below the heatmap further substantiate the observation that active domains generally contain a higher fraction of co-associated E-motifs per domain and less frequently pair with the D-motifs (additional data for motif counts is shown in Supplementary Figures S17 and S18). These analyses clearly show that individual domain categories are characterized by distinct motif signatures, suggesting that the establishment of active and accessible chromatin domains during MO development is likely co-determined by PU.1 itself and/or its co-associated factors.

## DISCUSSION

**Figure 9.**
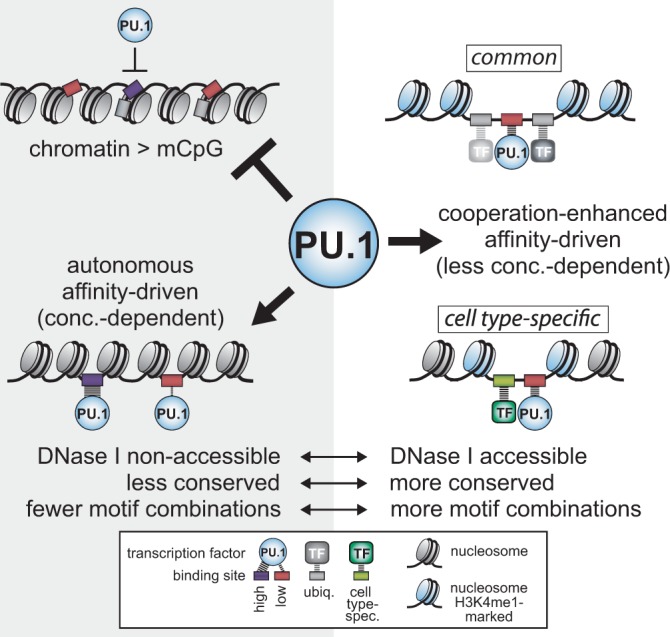
Schematic depicting the three classes of PU.1 consensus motifs.

Here, we have analyzed binding patterns of the common hematopoietic transcription factor PU.1 to reveal novel insights into prerequisites for DNA binding of this master regulator: overall, we can distinguish three major categories of consensus binding sites (summarized in Figure 9): (i) non-bound sites that mainly show low-binding affinity and reside in inactive chromatin. (ii) PU.1-bound sites that are DNase I inaccessible and represent ‘autonomous’ binding events preferentially at high-affinity sites. (iii) PU.1-bound mostly intermediate- and low-affinity sites that are DNase I accessible, and where binding is likely stabilized by cooperativeness with neighboring transcription factor binding sites. Increasing PU.1 concentration, which has previously been implicated in lineage-specific functions of PU.1 (16,35–39) reduces the binding affinity threshold, leading to a marked increase in autonomous binding sites and to a lower extent in cell type-specific sites.

Expression of the master regulator PU.1 is induced in early progenitor cells during hematopoiesis and retained at high levels in human monocytes/macrophages, myeloid dendritic cells, as well as granulocytes and to a lower level in early and pro-B-cell stages, erythrocyte–megakaryocyte progenitors or mast cells (40). Although PU.1 binds common sets of targets in these cell types, previous work identified a significant share of cell type-specific–binding events. The observed binding patterns were either attributed to cell type-specific transcription factor cooperativeness (7,8) or different PU.1 expression levels (16,39), which, however, explain only parts of the binding patterns. In fact, only a strikingly small fraction of all putative binding sites across the genome (<1%) is actually occupied by PU.1 in any of the cell types, and it is unclear how PU.1 is sequestered to the small and often cell stage-specific proportion of its binding sites. The DNA sequence represents a proximate determinant of transcription factor binding, and we initially addressed the aforementioned question by systematically analyzing sequence features of PU.1-bound sites. The PU.1 consensus PWM comprised >2500 different 12mers, which were found to bind PU.1 at variable frequencies. For example, 80% of all AAAGAGGAAGTG 12mers (809 instances) overlap with PU.1 ChIP-seq peaks within the non-repetitive genome, whereas only 1% of TAACTGGAAGTG 12mers (three instances) were considered occupied. The *in vivo* binding preference is reflected by the PWM motif log-odds score, which is a measure for the similarity of a given motif to the consensus PWM. Using microscale thermophoresis (21,29), we could demonstrate that the PU.1 PWM log-odds scores represent a surrogate for PU.1 motif-binding affinity. The comparison of motif features in different log-odds score (affinity) classes revealed a number of notable correlations. Most strikingly, we observed an inverse correlation between motif affinity and local DNA accessibility (as measured by DNase I cleavage frequency). High-affinity motifs were more often located in less accessible regions that also showed the lowest average vertebrate conservation. In contrast, highly accessible and more conserved regulatory modules were mostly populated by intermediate/low affinity motifs. Although we cannot directly infer functionality from this data, bound intermediate/low affinity motifs more likely reside in functionally important regions, as they are preferentially found in motif clusters that are also associated with ‘active’ epigenetic marks like H3K27ac and H3K4me1.

Even high-affinity motifs were not always associated with ChIP-seq tags, suggesting that some sites offer no or little opportunity for PU.1 to bind. DNA methylation, a principal mechanism of binding site selection for several other transcription factors, is not a major determinant of PU.1 binding—PU.1 binding was generally associated with local DNA demethylation, both in monocytes and macrophages, suggesting that PU.1 may participate in recruiting the DNA demethylation machinery to its binding sites. As a ‘master regulator’, PU.1 might actually be required to access relevant genomic sites, including cell type-specific enhancers, which are ‘silenced’ by DNA methylation in progenitor cells. This observation concurs with a recent study demonstrating the ability of transcription factors to induce local DNA demethylation (25). In line with such a role, the consensus sequence of PU.1 does not contain CpG dinucleotides within its core sequence, which may avoid a direct steric effect of DNA methylation on PU.1 binding. Insights from our comparison of bound and non-bound PU.1 elements across the genome, however, point to a role for higher-order chromatin structures in regulating PU.1 binding, regardless of the cell stage. Non-bound PU.1 elements were enriched in gene deserts or chromatin domains (defined as being flanked by the boundary transcription factor CTCF) that lack domain-wide monomethylation of histone H3 at lysine 4, a modification that correlates with transcriptional activity (41). Thus, a large proportion of non-bound PU.1 consensus motifs is located in inactive chromatin domains that are likely not or only partially accessible to PU.1. Exceptions most often include autonomous high-affinity motifs, which frequently show some degree of binding even in inactive chromatin domains. Interestingly, we also note that motif co-association patterns (the presence of PU.1 recognition sites close to other predicted binding sites) distinguish active and inactive domains, suggesting that the activity of chromatin domains is at least partially pre-determined by the underlying sequence context.

In contrast to factors like the glucocorticoid receptor (9), PU.1 binding is clearly not pre-determined by baseline chromatin accessibility patterns. Although DNase I non-accessible, autonomously bound motifs generally show little conservation (implying a lack of function), the ability of PU.1 to efficiently compete with nucleosomes at high-affinity sites may still be an important feature of its master regulator function. In this context, it will be interesting to clarify whether PU.1 binding simply relies on opportunity (on a stochastic basis) to access freely available DNA, or whether it can also bind or induce remodeling of nucleosome-associated DNA.

In conclusion, our analysis supports a hierarchical model for the regulation of PU.1 binding. The fact that some predicted high-affinity sites are not bound suggests that (higher order) chromatin structures can provide a first level of restriction. Motif-binding affinity generally presents the second layer of binding control that is fine-tuned by PU.1 expression levels and local binding site cooperativeness, which both lower the binding affinity thresholds.

## SUPPLEMENTARY DATA

Supplementary Data are available at NAR Online: Supplementary Tables 1 and 2, Supplementary Figures 1–18, Supplementary Methods and Supplementary Reference [42].

Supplementary Data
