# Supplementary material for: Mechanisms of in vivo binding site selection of the hematopoietic master transcription factor PU.1
Source: Nucleic Acids Res. 2013 May 8;41(13):6391–402. doi: 10.1093/nar/gkt355 (PMC3711439; doi:10.1093/nar/gkt355)
Supplement: Supplementary Data [file supp_41_13_6391__index.html]

Mechanisms of in vivo binding site selection of the hematopoietic master transcription factor PU.1 — Mechanisms of in vivo binding site selection of the hematopoietic master transcription factor PU.1 — Supplementary Data 

# Mechanisms of *in vivo* binding site selection of the hematopoietic master transcription factor PU.1

## Supplementary Data

files

**Files in this Data Supplement:**

- Supplementary Data - pdf file
