# Supplementary material for: Mechanisms of in vivo binding site selection of the hematopoietic master transcription factor PU.1
Source: Nucleic Acids Res. 2013 May 8;41(13):6391–402. doi: 10.1093/nar/gkt355 (PMC3711439; doi:10.1093/nar/gkt355)
Supplement: Supplementary Data [file supp_gkt355_nar-00573-m-2013-File011.pdf]

Supplementary data for

Mechanisms of *in-vivo* binding site selection of the hematopoietic master transcription factor PU.1

Thu-Hang Pham[1], Julia Minderjahn[1], Christian Schmidl[1], Helen Hoffmeister[2], Sandra Schmidhofer[1], Wei Chen[3], Gernot Längst[2], Christopher Benner[4,5], Michael Rehli[1]

[1]Department of Internal Medicine III,
University Hospital Regensburg, F.-J.-Strauss Allee 11, D-93042 Regensburg, Germany;

[2]Department of Biochemistry III, University of Regensburg, Universitätsstrasse 31, D-93053 Regensburg, Germany.

[3]Berlin Institute for Medical Systems Biology (BIMSB), Max-Delbrück-Centrum für Molekulare Medizin (MDC) Berlin-Buch, D-13092 Berlin, Germany

[4]Department of Cellular and Molecular Medicine, University of California San Diego, La Jolla, California, USA

[5]Integrative Genomics and Bioinformatics Core,
Salk Institute for Biological Studies, La Jolla, California, USA.

Supplement Index:

Supplementary Methods and References page 02-06
Summary or Supplementary Tables and Figures page 07
Supplementary Tables S1-2 page 08-09
Supplementary Figures S1-18 page 10-19

Supplementary Methods

Mass spectrometry analysis of bisulfite-converted DNA – Genomic regions for MassArray analyses were chosen that either indicated an epigenetic transition from HPC to monocytes (induction of H3K4me1) or the induced binding of transcription factors in combination with *de novo* H3K4me1 appearance during monocyte to macrophage differentiation. Primer design, sodium bisulfite conversion, amplification and MALDI-TOF mass spectrometry (MassARRAY Compact MALDI-TOF, Sequenom, San Diego, CA) were done as described. Methylation was quantified from mass spectra using the Epityper software (Sequenom, San Diego, CA). The following primers were used to generate amplicons from bisulfite treated DNA:

Primer name	Sequence 5'-3'
ADAP1_F	aggaagagagGGTGGAGAGGGAAGTGAATTTTTATAATT
ADAP1_R	cagtaatacgactcactatagggagaaggctTCCCTAATCCAAAACCAAAAACAAC
SMAD7_F	aggaagagagGAGGTTGGAGGTTATGAAGAGGTTT
SMAD7_R	cagtaatacgactcactatagggagaaggctTACTAACCACCCAATACAAACCCAC
RAB4A_F	aggaagagagGGGAGATTTTAGAGGGTTTTTAGGATT
RAB4A_R	cagtaatacgactcactatagggagaaggctAAAATAAACCTCCCCATTCCACTCT
NCRNA00111_F	aggaagagagAAAATTTGTAGGGTTGATTTGAAGT
NCRNA00111_R	cagtaatacgactcactatagggagaaggctATAAATCACAACAACACACTTTCCC
PIM3_F	aggaagagagGTTTGGTAGGTAGGGGTTTTGTTTT
PIM3_R	cagtaatacgactcactatagggagaaggctCCAAACTAAATCCTTCTCAACTCCTC
KCNE2_F	aggaagagagTGTGTGGTATTTTATTATGGTAGTTTTAGT
KCNE2_R	cagtaatacgactcactatagggagaaggctCACCCAAATAATCAATCCTCAAACC
VRK2_F	aggaagagagTTTTGGGAATTATGAGTGATAGATTTAGAA
VRK2_R	cagtaatacgactcactatagggagaaggctTCAAAATAAATTATACTTTCCTCCAATTT
TSSC1_F	aggaagagagTTTTTATGAGTGGGAGGTGTGAGTT
TSSC1_R	cagtaatacgactcactatagggagaaggctCCTCAAAATCCTACAAACTACCCAAAC
RSRC1_F	aggaagagagGGTTTTTTGTTATGTGAGAAAGATTTGTAG
RSRC1_R	cagtaatacgactcactatagggagaaggctCACACAACATTTCAAAACAACTCTC
CACNA1C_F	aggaagagagATGGAAGTTGAGAATTGAATTAATGAATGT
CACNA1C_R	cagtaatacgactcactatagggagaaggctCAAAACCAAAAAACATCCACAACAA
CORO1C_F	aggaagagagTTTTAGTGATAAGGGTTGGGTGTTG
CORO1C_R	cagtaatacgactcactatagggagaaggctCCCAATAAAAATTTTCCCAAAAAAAA
PLEKHA6_F	aggaagagagGGGTTTTTTTTGGGAGTTTTTTTT
PLEKHA6_R	cagtaatacgactcactatagggagaaggctACAATTAAACCATTAAACACCACAAC
OXSM_F	aggaagagagTTATTTTTAGGAATGAAAGGGGAAGG
OXSM_R	cagtaatacgactcactatagggagaaggctAAACAAACAACTCTTTCTCCAAATAAAACT
ST18_F	aggaagagagTTTTTTTGGATTTTGTTTAGGTAAG
ST18_R	cagtaatacgactcactatagggagaaggctAAACATTTCCCTACATCTTTATTTCAC
OSR1_F	aggaagagagTTTTGGTTTTAATTTAATGTTGTTATGTGG
OSR1_R	cagtaatacgactcactatagggagaaggctAAAAAAACCACAAATCTTTTTACAATTCC

Microscale Thermophoresis – The sequence of the full-length hPU.1 was amplified by PCR from pORF9-hSPI1 (InvivoGen San Diego, USA) and recombined into a modified pDM8 vector, encoding an N-terminal His-tag, using the Gateway technology (life technologies). The protein was expressed in Rosetta2(DE)pLysS (Novagen) and purified by Nickel affinity chromatography (Qiagen). Double-stranded DNA molecules were annealed from single-stranded, HPLC-purified oligonucleotides (Sigma-Aldrich). The annealing reaction (10 µl) was performed in 1x annealing buffer (20 mM Tris-HCl pH 7.4, 2 mM MgCl2, 50 mM NaCl) and comprised 20 µM of the Cy3-labeled oligonucleotide (upper strand) and 20.8 µM of the unlabeled oligonucleotide (lower strand). The annealing reaction was incubated for 15 min at 95°C in a thermoblock (peQLab) and afterwards allowed to slowly cool down to room

temperature over night. The annealing reaction was checked on an 8% native polyacrylamide gel which was analyzed on a fluorescence imager (FLA-5000, Fujifilm).

The binding assay was carried out using the Nanotemper Monolith NT.115 (initial settings: LED power: 90%, IR-laser power: 80%, 25°C). For each motif affinity measurement 16 reactions were prepared on ice (MST-buffer: 20 mM Tris-HCl pH 7.6, 1.5 mM MgCl2, 0,5 mM EGTA, 10% glycerol, 300 mM KCl, 10 mM DTT). The Cy3-labeled dsDNA oligo was always kept at a constant concentration of 50 nM. The unlabeled protein was titrated in a 1:1-dilution series starting with a concentration of 23 µM. Every binding assay comprised one control reaction without any protein. After loading the binding reactions into standard capillaries (NT.115) the mixture was incubated for 15 min at 25 °C in the Nanotemper before starting the measurement. The data was analyzed using the NT-analysis acquisition software (1.2.229), which plots a binding curve using the normalized fluorescence of the labeled dsDNA at different concentrations of the unlabeled protein. Each binding assay was performed twice and the mean value was calculated. For every single binding assay a maximum of three outlier values were eliminated. Four representative examples for thermophoresis curves are shown below.

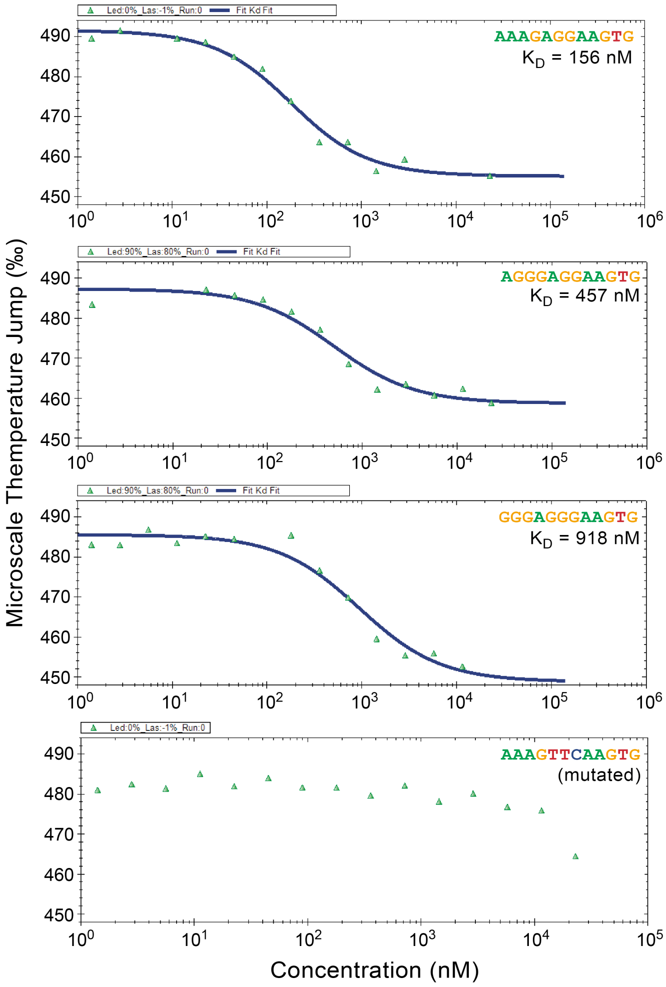

Figure Methods
Examples of thermophoresis curves obtained for four representative motifs. The top motif represents the motif with the highest PWM log-odds score, the bottom motif (mutated) contains a three nucleotide exchange in the core recognition site of PU.1 and shows no detectable binding.

ChIP-seq peak finding and annotation – Analysis of mapped ChIP-seq tags was performed using HOMER, which is freely available at http://biowhat.ucsd.edu/homer/. ChIP-Seq quality control and transcription factor peak finding, TSS annotation (based on GENCODE V13) and motif analysis were done essentially as described [1,2]. Genome Ontology annotation and ChIP-seq tag annotation of peak sets, or motif-centered regions was done using scripts provided by HOMER. Next generation sequencing data (either published or generated in this study) that were used in this study are listed below:

(A) Published sequencing data used in this study

Cell Type	Epitope/Method	Acc.	Total uniquely mapped tags (hg19)
Human CD133+ HPC	H3K4me1	GSM317596	9,249,040
	H2AZ	GSM317592	8,239,063
	PU.1	GSM638310, GSM638310	8,556,878
Human CD34+ HPC	DNaseI	GSM493384, GSM493387, GSM530660, GSM530664, GSM595917, GSM595918	178,649,699
Human blood monocytes	H3K4me1	GSM785492	22,731,462
	H2AZ	GSM785493	53,730,274
	H3K27ac	GSM785494	50,683,332
	PU.1	GSM785495	14,586,253
	H3K4me1	GSM1003535	24,054,372
	H3K4me2	GSM1003516	47,885,749
	H3K4me3	GSM1003536	31,570,886
	H2AZ	GSM1003548	32,467,943
	H3K27ac	GSM1003559	34,699,392
	H3K9ac	GSM1003515	25,168,970
	H3K9me3	GSM1003538	20,505,232
	H3K27me3	GSM1003564	40,731,147
	Input	GSM1003575	38,836,156
	DNaseI	GSM701541, GSM701503, GSM665840	67,361,132
Human monocyte-derived macrophage	H3K4me1	GSM785498	22,731,462
	H2AZ	GSM785499	54,740,528
	H3K27ac	GSM785500	50,683,332
	PU.1	GSM785501	15,697,524
	IgG	GSM785497	13,435,897
Osteoblasts	H3K4me1	GSM733704	41,436,351
	Input	GSM733697	41,742,824
Liver	H3K4me1	GSM537706, GSM621654, GSM669972	72,718,289

(B) High-throughput sequencing data generated in this study
(Sequencing data is available at GEO, accession no. GSE43098)

Cell Type	Epitope	Donor	Total uniquely Mapped tags (hg19)
Human blood monocytes	CTCF	E	5,653,880
	mCpG (MCIp)	E	8,208,789
Human monocyte-derived macrophage	CTCF	E	5,500,643

De novo motif searches – Motif enrichment in transcription factor peak sets was done using HOMER by comparing sequences of cell type-specific peaks (+/- 100 bp) to 50,000 randomly selected genomic fragments of the same size, matched for GC content and autonormalized to remove bias from lower-order oligo sequences. Due to the numerous enrichment tests made during the motif discovery procedure and the vast search space, corrections for multiple hypothesis testing must be carried out empirically by randomizing the target and background assignments and repeating the motif discovery procedure. One hundred randomizations (which were performed for each individual motif search) failed to yield motifs with enrichment *P*-values less than 1e-19, implying the false discovery rate for motifs with a *P*-value less than 1e-19 reported in this study is < 1%. Motif enrichment around bound motifs (+/- 100 bp) was done by comparing motif-centered regions with non-overlapping, GC-matched, and autonormalized regions centered on non-bound motifs. In either case, motif enrichment is calculated using the cumulative hypergeometric distribution by considering the total number of target and background sequence regions containing at least one instance of the motif.

Known motif analyses – The *de novo*-derived PU.1 motif with the broadest coverage (MAC peak derived) was used for all downstream analyses unless noted otherwise.

MAC-derived consensus PWM:

CACTTCCTCWTW

HOMER determined log-odds score threshold: 6.751640

A	C	G	T
0.177	0.447	0.200	0.178
0.672	0.058	0.178	0.093
0.019	0.627	0.271	0.084
0.032	0.009	0.009	0.951
0.012	0.012	0.013	0.964
0.011	0.958	0.007	0.025
0.005	0.837	0.005	0.154
0.068	0.305	0.144	0.485
0.073	0.509	0.152	0.267
0.349	0.178	0.062	0.413
0.230	0.224	0.108	0.440
0.275	0.205	0.104	0.418

Reannotation of the PWM to the human genome (hg19, either total or repeat-masked) was done using the scanMotifGenomeWide.pl script contained in the HOMER suite. HPC, MO, and MAC PU.1 ChIP-seq tags were counted around all motif instances (+/- 100 bp) across the repeat-masked human genome to determine non-bound PU.1 motifs (no ChIP-seq tag within the 200-bp window) across the non-repetitive genome. To determine the total set of bound PU.1 motifs, all bound PU.1 motif instances from HPC, MO and MAC were merged using bedTools. Extraction of log-odds scores for individual motifs or peaks was done using the annotatePeaks.pl program (provided by HOMER), which returns the highest scoring motif position as well as log-odds scores for each peak/region. Sequences were extracted using homerTools (provided by HOMER).

Analyses of published DNase I sequencing data – Published HPC and MO DNase-Seq raw sequencing data (for accession no. see Table S1) from several available donors (corresponding data

sets combined) were mapped to hg19 using Bowtie [3] and analysed (at nucleotide resolution) using HOMER. To define DNase I accessible regions, we searched for peaks in small windows (10bp) that were stitched together if less than 10 bp apart using the region option of the findPeaks program. BedTools [4] were used to separate motif and peak sets (200 bp size) into DNase I region overlapping and non-overlapping ones.

Identification of PU.1 motif pairs and clusters – BedTools were used to separate single PU.1 motifs from motifs that occur in close neighborhood (less than 100 bp apart). BedTools were also used to remove motif pairs or clusters residing in the repetitive genome.

Analyses of CTCF-flanked domains – CTCF binding sites were determined from ChIP-seq data generated in this study. For analyses on domain level, we focused on regions flanked by two CTCF sites either in MO or MAC that were ≥ 10,000 bp apart. Analyses were also restricted to autosomes to avoid including gender-dependent differences. To determine domain 'activities', H3K4me1 tags were counted into individual domains and normalized for region size. For downstream analyses, domains were either binned according to their size-normalized domain activities, or collected based on their cell type-specific activites (>4-fold difference). Motif occurrences for transcription factors were mapped using HOMER. Overlaps between domains or PU.1 consensus site-surrounding regions were detected using BedTools.

Supplementary References

1. Heinz S, Benner C, Spann N, Bertolino E, Lin YC, et al. (2010) Simple combinations of lineage-determining transcription factors prime cis-regulatory elements required for macrophage and B cell identities. Mol Cell 38: 576-589.
2. John S, Sabo PJ, Thurman RE, Sung MH, Biddie SC, et al. (2011) Chromatin accessibility pre-determines glucocorticoid receptor binding patterns. Nature genetics 43: 264-268.
3. Langmead B (2010) Aligning short sequencing reads with Bowtie. Current protocols in bioinformatics / editoral board, Andreas D Baxevanis [et al] Chapter 11: Unit 11 17.
4. Quinlan AR, Hall IM (2010) BEDTools: a flexible suite of utilities for comparing genomic features. Bioinformatics 26: 841-842.

Summary of Supplementary Tables and Figures

**Table S1**	Complete results of the microscale thermophoresis measurements for 75 selected sequences included in the PU.1 PWM.
**Table S2**	Characteristics of chromatin domain categories.
**Figure S1**	Dynamics of PU.1 binding during HPC to monocyte differentiation.
**Figure S2**	Genome Ontology enrichment analysis for bound and non-bound PU.1 motifs
**Figure S3**	Distribution of epigenetic marks at bound and non-bound PU.1 consensus sequences.
**Figure S4**	Relationship between transcription factor binding and DNA methylation.
**Figure S5**	Motif composition around bound vs. non-bound PU.1 consensus sites or around PU.1 peaks not recognized by the consensus PU.1 motif.
**Figure S6**	Distribution of motif log-odds scores at PU.1 bound regions for three cell stages and alternative PWM.
**Figure S7**	Comparison of motif log-odds scores with signal intensity Z scores from protein binding microarray (PBM) experiments.
**Figure S8**	Characterization of homotypic PU.1 motif clusters.
**Figure S9**	Bound PU.1 motifs with differentiation-dependent DNase I accessibility changes.
**Figure S10**	PU.1 motifs in gene deserts.
**Figure S11**	Expression correlation in CTCF-flanked domains contingent of their H3K4me1 level.
**Figure S12**	Motif distribution in CTCF-flanked domains contingent of their H3K4me1 level.
**Figure S13**	Distribution of motif-associated PU.1 tag counts in CTCF-flanked domains contingent of their H3K4me1 level.
**Figure S14**	Motif score distribution and DNAse I accessibility in CTCF-flanked domains contingent of their H3K4me1 level.
**Figure S15**	Cell type-specific domain activities in MO, MAC and HPC.
**Figure S16**	Motif analyses in domains with differential activity between MO and liver.
**Figure S17**	Enrichment of PU.1 co-associated transcription factor consensus sites in domains showing cell type-specific activity
**Figure S18**	Distribution of PU.1 motifs across domain categories.

Table S1
Microscale thermophoresis-derived K_D values for selected PU.1 motifs

Oligo sequence	Log-odds score	% bound	K_D Exp.1	K_D Exp.2	K_D MW	Δ
acgtCACTTCCTCTTTacgt	10.68	80.50	156	173	164.5	17
acgtCACTTCCTCATTacgt	10.51	68.13	254	290	272	36
acgtCACTTCCCCTTTacgt	10.22	71.04	484	423	453.5	61
acgtCACTTCCTCTATacgt	10.03	38.27	371	455	413	84
acgtCACTTCCTCTCTacgt	10.01	38.75	258	281	269.5	23
acgtGACTTCCTCTTTacgt	9.88	63.76	206	175	190.5	31
acgtCAGTTCCTCTTTacgt	9.84	57.70	200	245	222.5	45
acgtCACTTCCCCTTAacgt	9.80	33.64	432	450	441	18
acgtCACTTCCTCTCAacgt	9.59	24.61	643	710	676.5	67
acgtCACTTCCTGTTTacgt	9.47	57.23	183	167	175	16
acgtCACTTCCTCAAAacgt	9.45	49.08	245	240	242.5	5
acgtCACTTCCTTTCTacgt	9.36	8.13	540	635	587.5	95
acgtCACTTCCTCTACacgt	9.32	10.90	427	710	568.5	283
acgtCACTTCCTCTGTacgt	9.28	18.36	524	791	657.5	267
acgtGACTTCCTCTCTacgt	9.20	21.70	301	346	323.5	45
acgtAACTTCCTCATAacgt	9.17	1.76	417	472	444.5	55
acgtCACTTCCTCCCTacgt	9.17	27.11	457	483	470	26
acgtCACTTCCTCAACacgt	9.15	13.91	506	399	452.5	107
acgtCAGTTCCTCTTCacgt	9.13	22.88	592	599	595.5	7
acgtAACTTCCCCATTacgt	9.12	4.90	223	244	233.5	21
acgtGAGTTCCTCTTTacgt	9.04	39.02	290	305	297.5	15
acgtGACTTCCTCATCacgt	9.00	18.21	526	537	531.5	11
acgtCACTTCCCCAAAacgt	8.98	30.39	311	414	362.5	103
acgtAACTTCCTCAATacgt	8.94	2.42	424	501	462.5	77
acgtCACTTCCGCTTCacgt	8.76	29.75	578	648	613	70
acgtAAGTTCCTCATTacgt	8.75	4.13	560	419	489.5	141
acgtCTCTTCCTCTTTacgt	8.70	24.78	661	777	719	116
acgtTACTTCCTTTTAacgt	8.70	0.50	436	407	421.5	29
acgtGACTTCCTGTTTacgt	8.67	41.73	388	334	361	54
acgtCAGTTCCTGTTTacgt	8.63	35.88	520	597	558.5	77
acgtCAGTTCCTCAAAacgt	8.61	19.12	670	528	599	142
acgtAACTTCCTCCTAacgt	8.50	1.43	502	425	463.5	77
acgtAACTTCCCCAATacgt	8.47	0.82	640	582	611	58
acgtCACTTCCTCCCCacgt	8.45	20.81	623	757	690	134
acgtTGCTTCCTCTTTacgt	8.43	10.10	368	460	414	92
acgtCCCTTCCTCTTTacgt	8.23	16.02	647	718	682.5	71
acgtCACTTCCTGCTAacgt	8.21	5.42	738	850	794	112
acgtCGCTTCCCCTTCacgt	8.18	13.55	951	835	893	116
acgtCGCTTCCTCTCCacgt	7.97	8.90	936	1030	983	94
acgtCAGTTCCTTAAAacgt	7.96	6.83	857	760	808.5	97
acgtCACTTCCTAAATacgt	7.92	7.34	546	693	619.5	147
acgtCACTTCCGCCTCacgt	7.91	11.67	752	561	656.5	191
acgtCACTTCCTCTGGacgt	7.89	11.76	677	966	821.5	289
acgtCACTTCTCTTTTacgt	7.88	11.51	683	857	770	174
acgtCATTTCCTCCTTacgt	7.83	3.72	832	824	828	8
acgtCACTTCCTCCAGacgt	7.80	5.67	628	640	634	12
acgtCGCTTCCTCCTCacgt	7.80	5.86	664	850	757	186
acgtCACTTCTGCTTTacgt	7.77	42.66	597	604	600.5	7
acgtCAGTTCCTCCTGacgt	7.61	3.78	1040	818	929	222
acgtCTCTTCCCCTCTacgt	7.57	1.10	1670	2560	2115	890
acgtCACTTCCTTGTCacgt	7.43	5.95	806	765	785.5	41
acgtGCCTTCCTCTTTacgt	7.43	14.29	939	835	887	104
acgtCACTTCCATTCTacgt	7.40	-	3310	5880	4595	2570
acgtCTCTTCCTTTCTacgt	7.38	0.39	1480	1920	1700	440
acgtCACTTCCCTCCCacgt	7.34	7.45	918	1110	1014	192
acgtCGTTTCCTCTTTacgt	7.34	15.28	742	597	669.5	145
acgtGAGTTCCTTTAAacgt	7.33	-	1750	1580	1665	170
acgtCACTTCCCCAGGacgt	7.25	5.30	1180	951	1065.5	229
acgtAACTTCTTCCTTacgt	7.22	-	1630	1610	1620	20
acgtCGCTTCCTTCCTacgt	7.19	3.48	1900	1890	1895	10
acgtATCTTCCTCATAacgt	7.19	-	829	752	790.5	77
acgtCTCTTCCTCCCTacgt	7.19	2.28	1310	2570	1940	1260
acgtCACTTCCACAGTacgt	7.14	-	2040	1820	1930	220
acgtGACTTCCACAATacgt	7.10	0.90	1050	1400	1225	350
acgtTTCTTCCTCTTCacgt	7.07	1.16	843	900	871.5	57
acgtCAGTTCTCTTTTacgt	7.04	3.77	2020	1980	2000	40
acgtGACTTCCTTCACacgt	7.03	-	1430	1060	1245	370
acgtGACTTCTGCTTTacgt	6.97	25.84	802	853	827.5	51
acgtCAGTTCTGCTTTacgt	6.94	17.45	895	923	909	28
acgtATCTTCCTCACTacgt	6.93	0.18	1970	2070	2020	100
acgtTGCTTCCTCCCTacgt	6.92	2.84	3020	1380	2200	1640
acgtAATTTCCTCATCacgt	6.86	-	959	1210	1084.5	251
acgtAACTTCTTCAAAacgt	6.83	-	1230	1810	1520	580
acgtCTCTTCCTTAAAacgt	6.82	1.36	1150	1150	1150	0
acgtCAGTTCTGCATTacgt	6.77	4.87	1120	1260	1190	140

Table S2
Characteristics of chromatin domain categories

	Number of domains in category	Total size of domains (Mb)	Total motif count in domains (in thousands)
**Gene deserts**	453	529	182
**H3K4me1 TC/bp 0.00 in MO**	10815	2111	829
**H3K4me1 TC/bp 0.01 in MO**	5495	486	221
**H3K4me1 TC/bp 0.02 in MO**	1029	59	31
**H3K4me1 TC/bp 0.03 in MO**	254	10	6
**H3K4me1 TC/bp 0.04 in MO**	157	4	3
**MO-specific (vs. Liver)**	597	34	17
**Liver-specific**	482	77	29
**MO-specific (vs. OB)**	1170	75	36
**OB-specific**	670	114	49

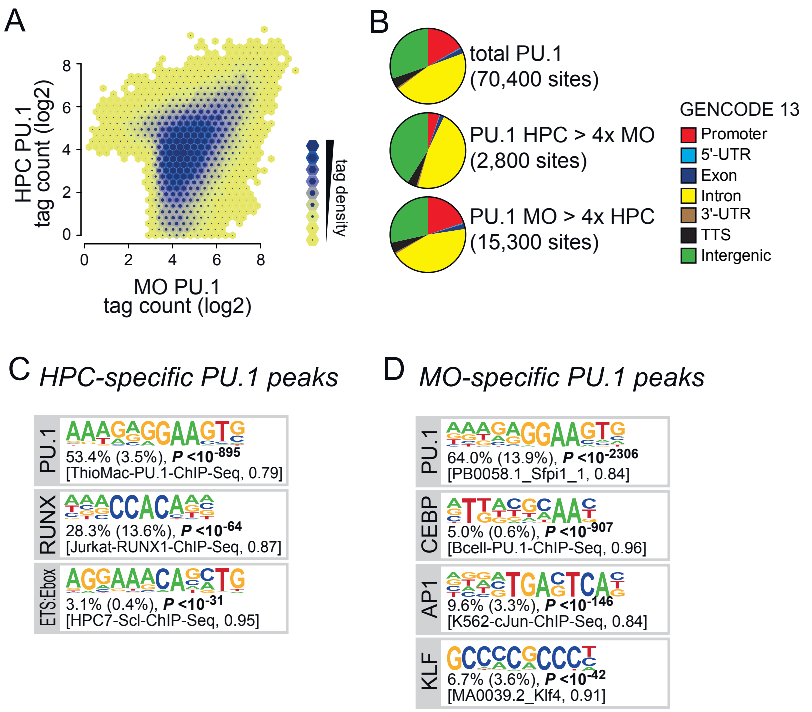

Figure S1

Dynamics of PU.1 binding during HPC to monocyte differentiation. (A) PU.1 ChIP-Seq tag counts for peak regions are compared between human CD133+ hematopoietic progenitor cells (HPC) and monocytes (MO) in a hexbin density plot. The colors represent the relative density of peaks in each location within the density plot. (B) Genomic distribution of total and cell stage-specific (at least four-fold different) PU.1 bound regions relative to GENCODE Genes V13. (C) *De novo* identified sequence motifs associated with PU.1 peak regions in HPC or MO. The fraction of PU.1 bound regions (200 bp) containing at least one motif instance, the expected frequency of the motif in random sequences (in parentheses) as well as *P* values (hypergeometric) for the overrepresentation are given below each motif. At the bottom of each panel, the best matching known motif is given along with its similarity score.

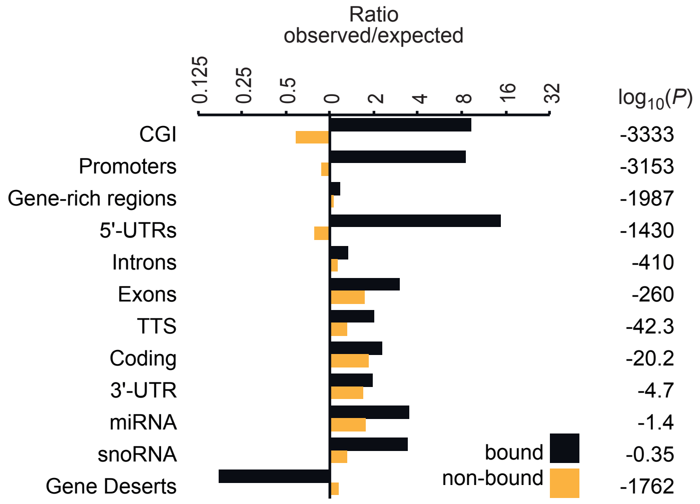

Figure S2

Genome ontology enrichment analysis for bound and non-bound PU.1 motifs. The bar chart shows log_2-ratios of observed versus expected annotation frequencies (based on RefSeq) across the human genome. Adjusted enrichment *P*-values (hypergeometric, log_{10}) for comparisons between bound and non-bound motifs are given on the right.

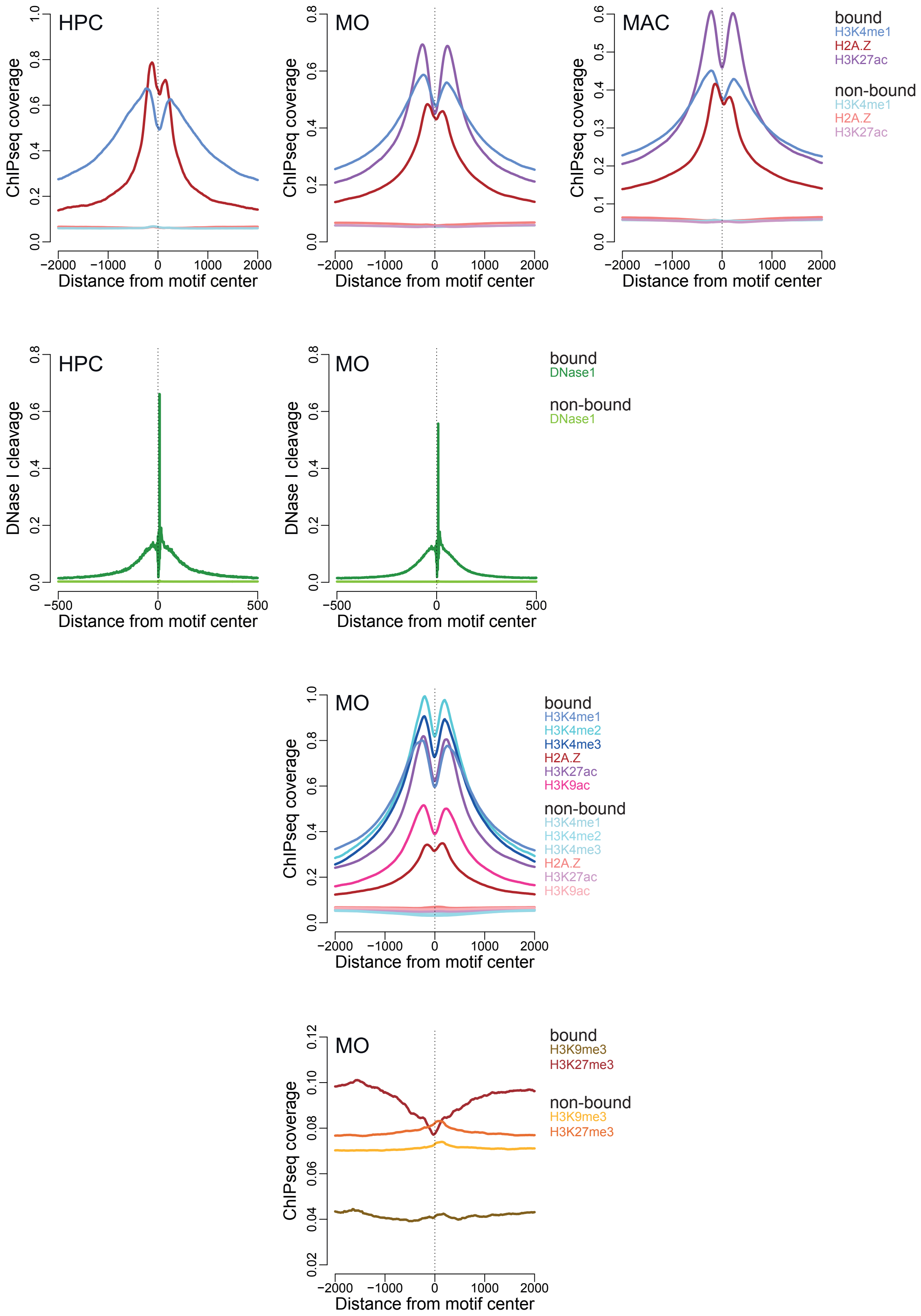

Figure S3

Distribution of epigenetic marks at bound and non-bound PU.1 consensus sequences. Histograms for genomic distance distributions of the indicated epigenetic data sets centered across bound or unbound PU.1 consensus sites across a 4-kb (or 1-kb) genomic interval. The top panel includes data from (Pham et al., 2012), the remaining data was generated by the ENCODE or the Roadmap Epigenomics projects (high-throughput sequencing data sets used in this study are listed in Table S1).

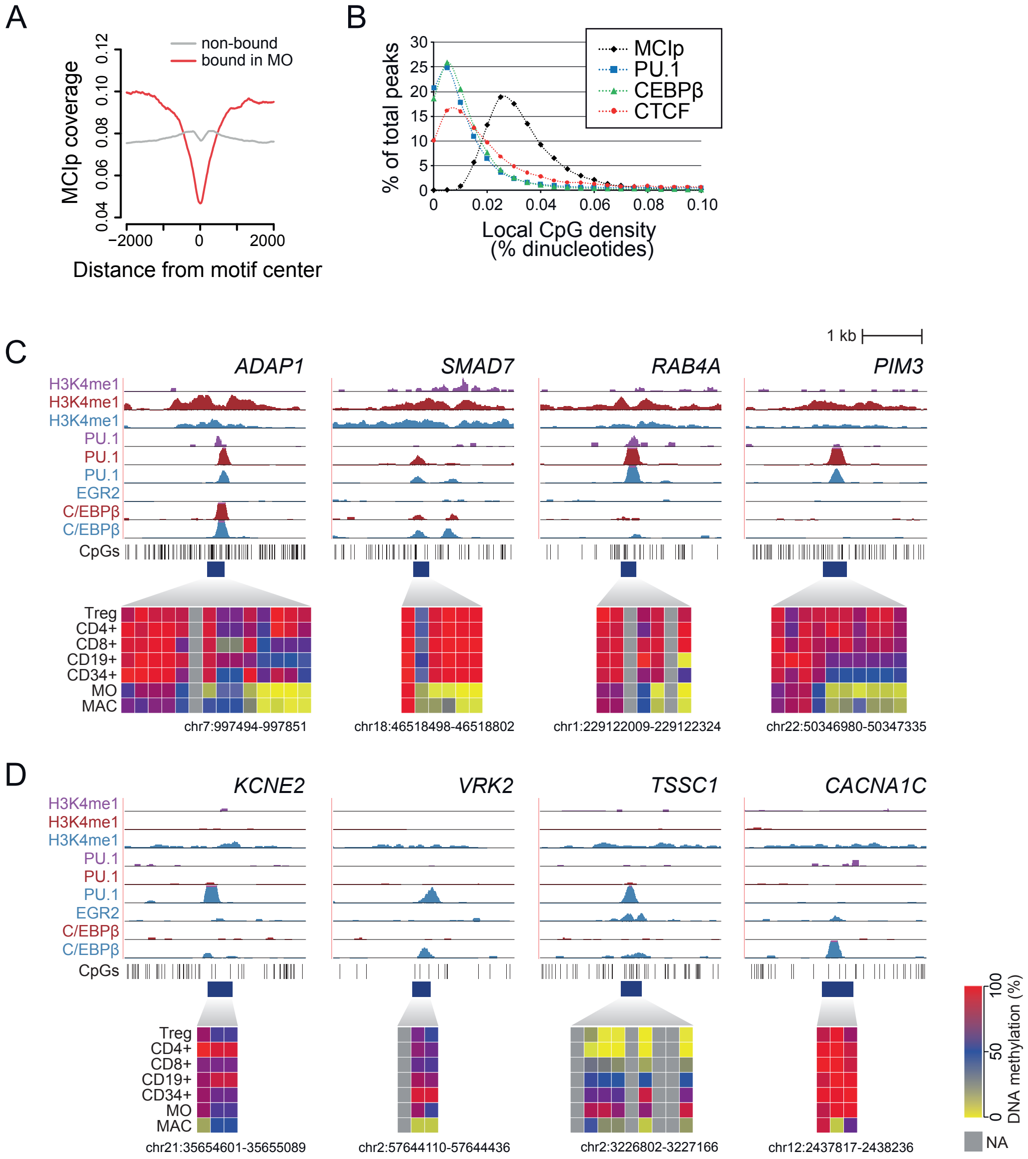

Figure S4

Relationship between transcription factor binding and DNA methylation. (A) Histograms for genomic distance distributions of MCIp tag counts (DNA methylation) centered across bound or non-bound PU.1 consensus sites across a 4 kb genomic region. (B) Histogram showing the Overlap between transcription factor bound sites and regions of high/intermediate CpG density. Peak numbers are plotted against binned CpG densities for DNA methylation enrichment data (MCIp-seq) and transcription factor ChIP-Seq experiments. (C-D) Additional examples for transcription factor-bound, promoter-distal regions that showed the specific absence of H3K4me1 in HPC (C) or macrophage-specific transcription factor binding (D) were subjected to DNA methylation analysis. Indicated ChIP-Seq tracks for HPC (purple), monocytes (red) and macrophages (blue) are shown for each region in the top panels. Positions of CpG dinucleotides are indicated as vertical lines below the tracks and regions analyzed by MALDI TOF MS of bisulfite-converted DNA from the indicated blood cell types are indicated by the dark blue boxes. Heat maps depict the methylation status of individual CpGs from red (100%) over blue (50%) to yellow (0%) with each box representing a single CpG. Data of at least three independent donors were averaged.

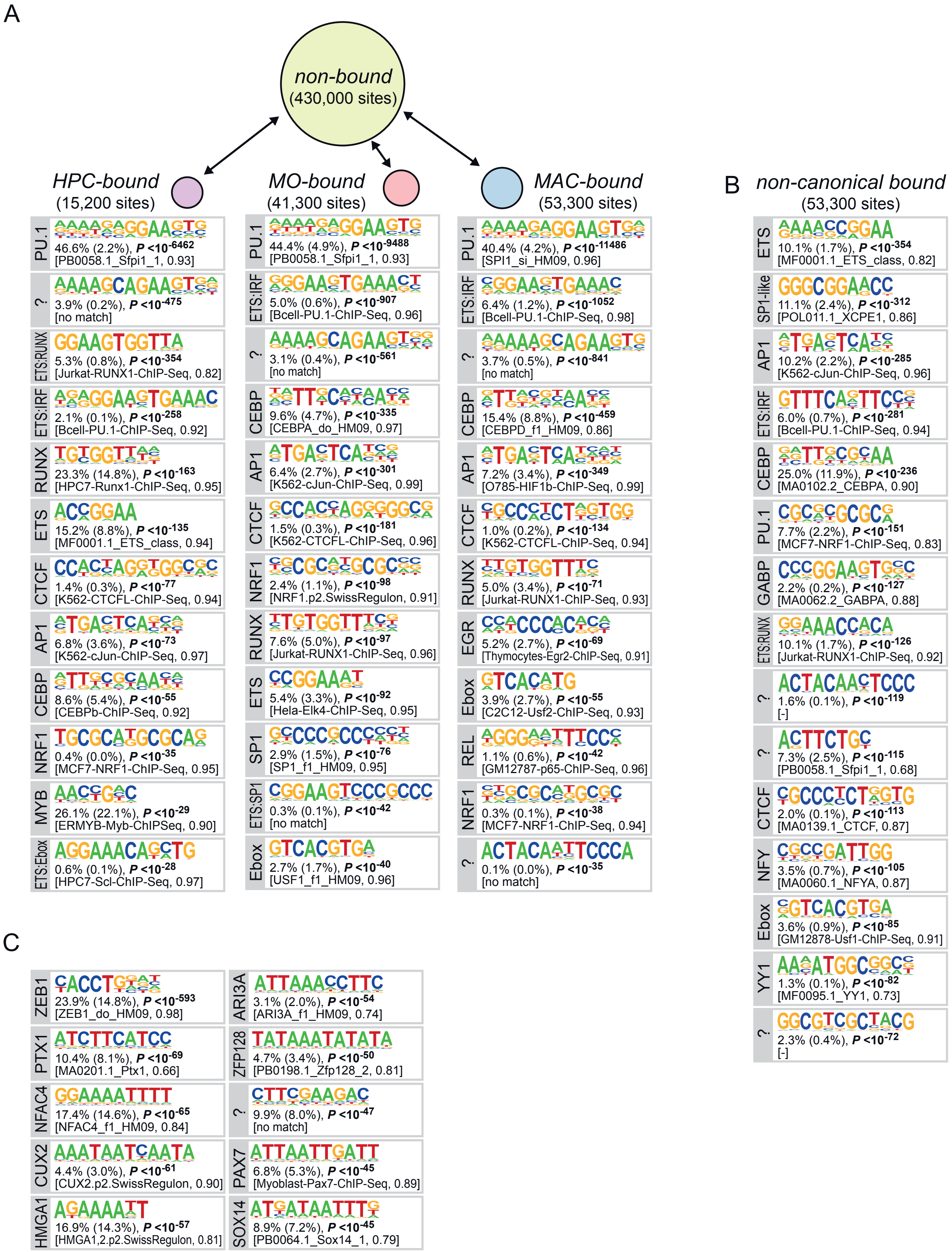

Figure S5

Motif composition around bound vs. non-bound PU.1 consensus sites or around PU.1 peaks not recognized by the consensus PU.1 motif. *De novo* derived sequence motifs associated with (A) bound peak regions (motif-centered) in HPC, MO and MAK compared to non-bound, motif-centered regions, (B) non-canonical PU.1 peak regions (not recognized by the consensus PWM) compared to non-bound, motif-centered regions and (C) non-bound, motif-centered regions compared to all bound peak regions (motif-centered) in HPC, MO and MAK. The fraction of regions (200 bp windows) containing at least one motif instance, the expected frequency of the motif in bckground sequences (in parentheses) as well as *P* values (hypergeometric) for the overrepresentation of each motif are provided below each motif. At the bottom of each panel, the best matching known motif is given along with its similarity score. In (C), only motifs are shown that show elevated levels of vertebrate conservation compared to neighboring sequences.

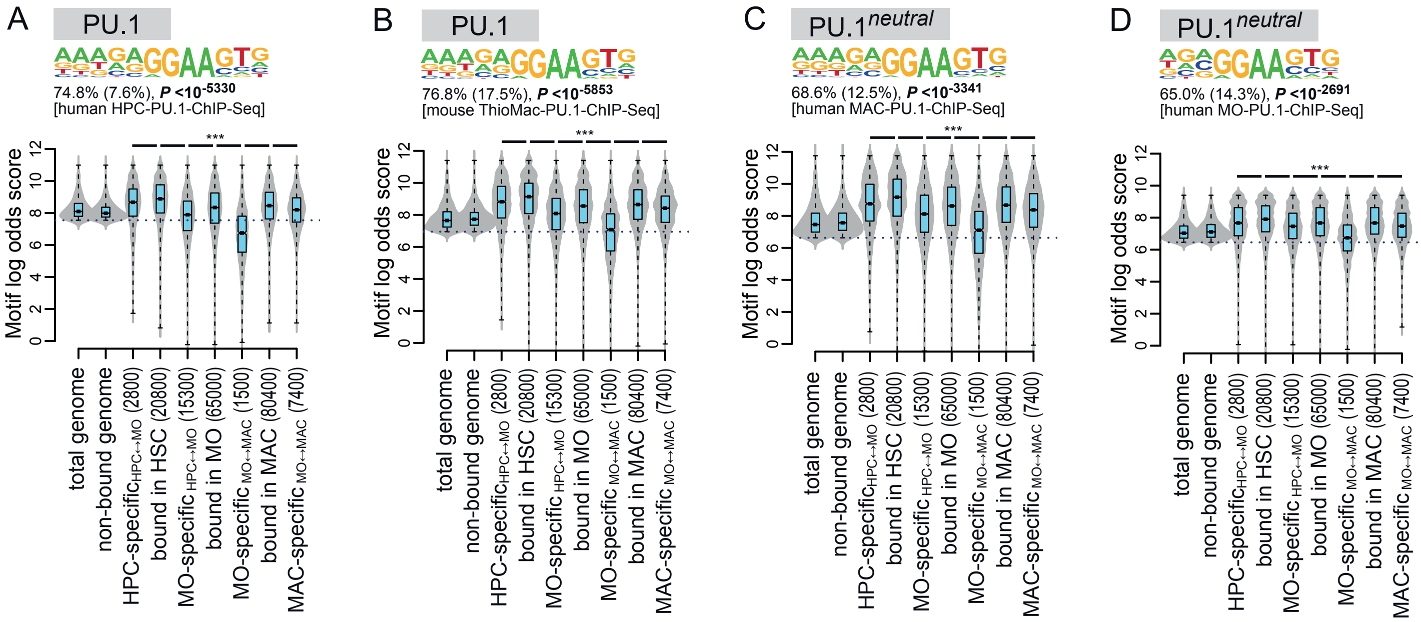

Figure S6

Distribution of motif log-odds scores at PU.1 bound regions for three cell stages and alternative PWM. (A-D) Combined bean & box plots showing the distribution of motif log-odds scores of annotated PU.1 motifs (white boxes) or best scoring motifs within total (blue boxes) or cell type-specific peaks (light blue boxes). (A) corresponds to the motif *de novo* extracted from human HPC PU.1 peaks, (B) corresponds to a motif *de novo* extracted from mouse peritoneal macrophage PU.1 peaks. In (C,D), motifs *de novo* derived motifs from human macrophage and monocyte PU.1 peaks were used, which were generated using normalized motif frequencies to correct for the depletion of CpG containing motifs. Solid bars of boxes display the interquartile ranges (25–75%) with an intersection as the median; whiskers, max/min values. Significantly different motif score distributions in pairwise comparisons are indicated (*** P < 0.001, Mann-Whitney U test, two-sided). The log odds score representing the motif detection threshold is indicated by the horizontal dotted line. The motif logos are shown on top of each plot along with the fraction of PU.1 bound regions (200 bp) containing at least one motif instance, the expected frequency of the motif in random sequences (in parentheses) as well as P values (hypergeometric) for the overrepresentation

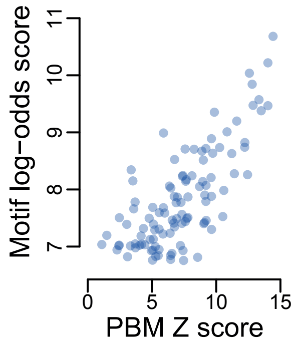

Figure S7

Comparison of motif log-odds scores with signal intensity Z scores from protein binding microarray (PBM) experiments. Published PBM data for the DNA binding PU.1 ETS domain and all possible 8-mers was used to compare PBM signal intensity Z scores, which represent a measure of protein affinity, with motif log-odds scores. To adjust for size differences between both measures, we focussed on the central 8-mers in our PWM (NNGGAANN). If several 12-mers of the original PWM overlapped a core PWM 8-mer, the highest log-odds score was assigned to it. The scatterplot shows a good agreement between both measures (coefficient of determination R^2=0.59).

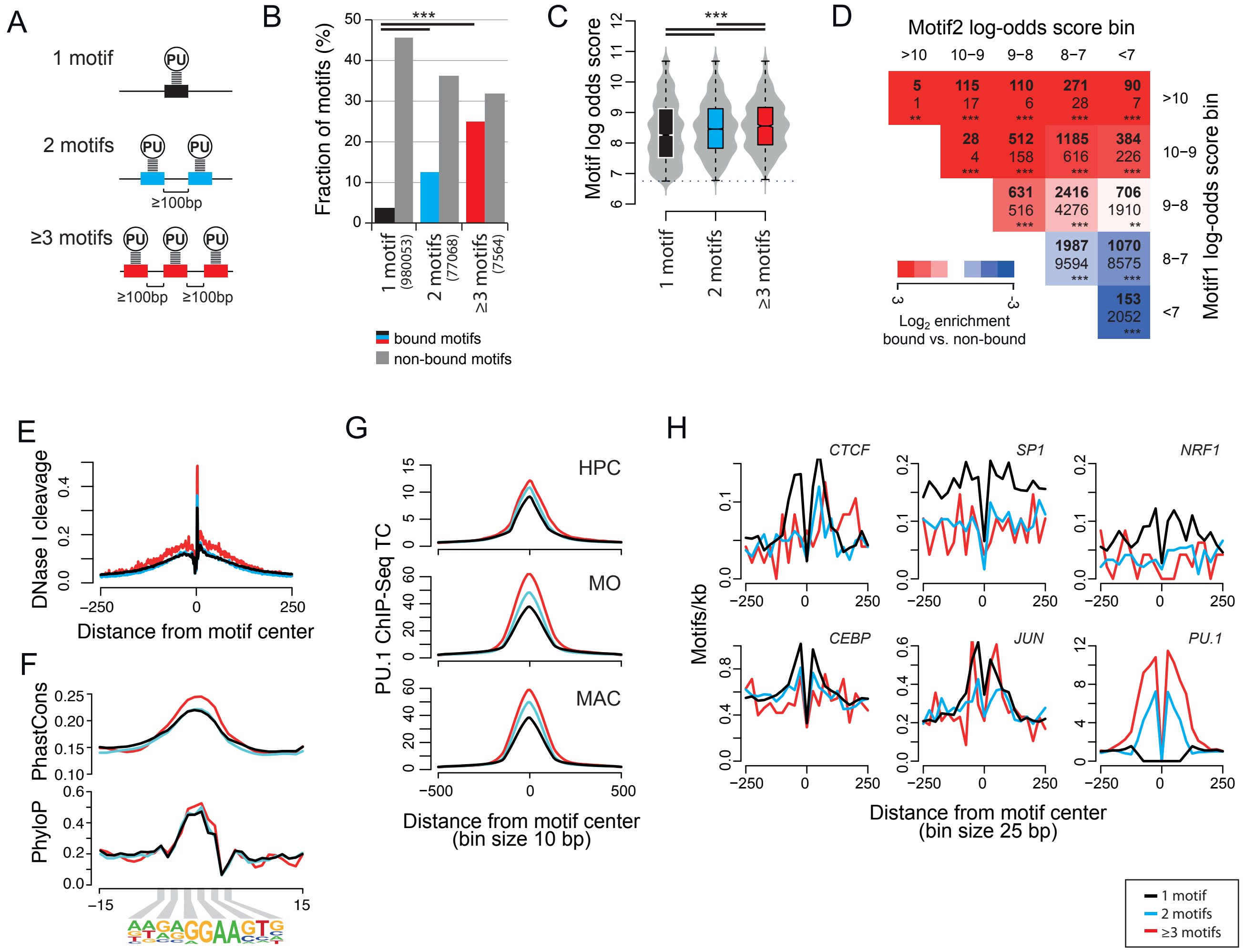

Figure S8

Characterization of homotypic PU.1 motif clusters. (A) Non-repetitive PU.1 motif occurences were segregated according to neibouring motif frequency into single PU.1 motifs (1 motif), pairs of two neighboring motifs (2 motifs), and clusters of three or more motifs (≥3 motifs) seperated by less than 100bp. (B) Bar chart showing the fractions of bound and non-bound motifs, motif pairs or motif clusters. Clusters and pairs are more frequently bound than single motifs (*** $P < 10^{-300}$, hypergeometric test). (C) Combined bean & box plot showing the distribution of motif log-odds scores of single motifs (black), as well as the highest scoring motifs of pairs (blue) and clusters (red). Solid bars of boxes display the interquartile ranges (25–75%) with an intersection as the median; whiskers represent max/min values. Motif score distributions in pairwise comparisons are highly significant (*** $P < 0.001$, Mann-Whitney U test, two-sided). The detection threshold is indicated by the dotted line. (D) Heatmap illustrating the relative and absolute frequencies of motif log-odds score combinations in motif pairs. Motif 1 represents the motif with the higher score. Numbers in bold indicate the absolute number of bound pairs of a given combination. The corresponding number of non-bound pairs is given below. Asterixs indicate that the bound fraction is either significantly enriched or depleted relative to non-bound (*** $P < 0.001$, ** $P < 0.01$, hypergeometric test). Enrichment ratios are indicated by coloring. (E) Histogram for the distribution of DNase I cleavage sites in MO centered across single motifs (black), as well as the highest scoring motifs of pairs (blue) and clusters (red) across a 500 bp genomic interval. (F) Histograms showing average per-nucleotide vertebrate conservation (PhastCons & PhyloP) surrounding single motifs (black), as well as the highest scoring motifs of pairs (blue) and clusters (red). (G) Histograms for the distribution of PU.1 Chip-seq tag counts (TC) in HPC, MO and MAC centered across single motifs (black), as well as the highest scoring motifs of pairs (blue) and clusters (red) across a 1kb genomic interval. (H) Histograms showing the distribution of indicated consensus motifs around PU.1 motifs as a function of motif frequency.

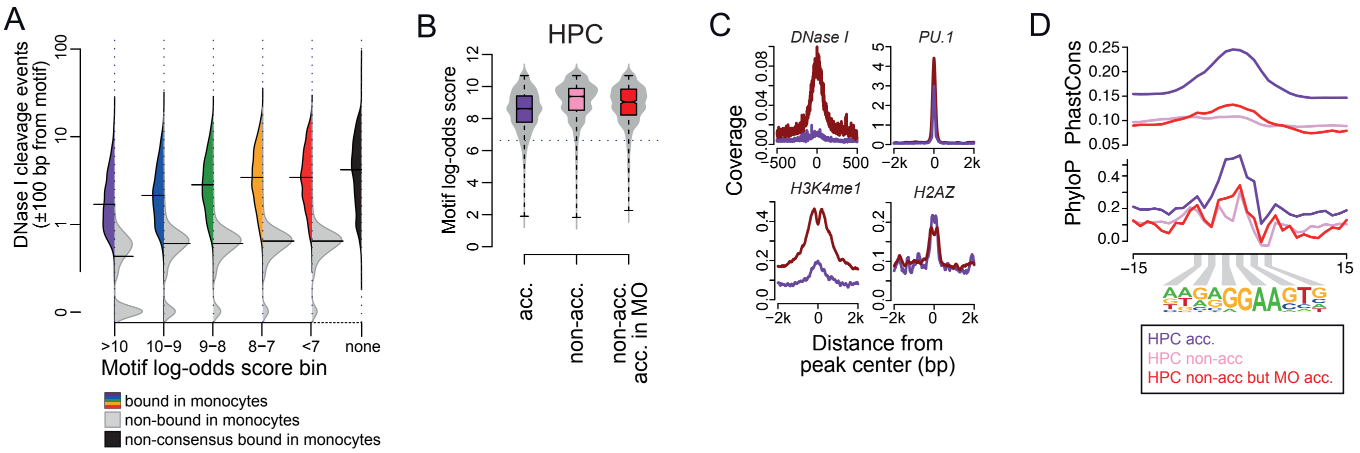

Figure S9

Bound PU.1 motifs with differentiation-dependent DNase I accessibility changes. (A) Bean plots showing the distribution of DNase I cleavage frequency around HPC PU.1 bound (consensus site: colored filling, non-consensus sites: black filling) and non-bound motifs (gray filling) depending on motif score classes. DNase I cleavage events (at nucleotide resolution, tag counts normalized to 10^7) were counted in a 200-bp window around each motif. Horizontal bars mark the median of each distribution. DNase I cleavage data (representing four independent donors) were originally generated by the ENCODE or the Roadmap Epigenomics projects (for accession nos. see the Supplementary Methods).(B) Combined bean & box plot showing the distribution of motif log-odds scores of PU.1 motifs in that are bound and accessible in HPC (purple), bound and non-accessible in HPC (rose) or bound and non-accessible in HPC but accessible in MO (red). Solid bars of boxes display the interquartile ranges (25–75%) with an intersection as the median; whiskers, max/min values. Significantly different motif score distributions in pairwise comparisons are indicated (*** $P < 0.001$, Mann-Whitney U test, two-sided). (C) Histograms for genomic distance distributions of the indicated sequencing data sets centered across PU.1 consensus sites that are bound and non-accessible in HPC but accessible in MO across a 4-kb (or 1-kb) genomic interval. HPC data is in purple and MO data in dark red. (D) Histogram showing average per-nucleotide vertebrate conservation (PhastCons) surrounding PU.1 motifs in that are bound and accessible in HPC (purple line), bound and non-accessible in HPC (rose line) or bound and non-accessible in HPC but accessible in MO (red line).

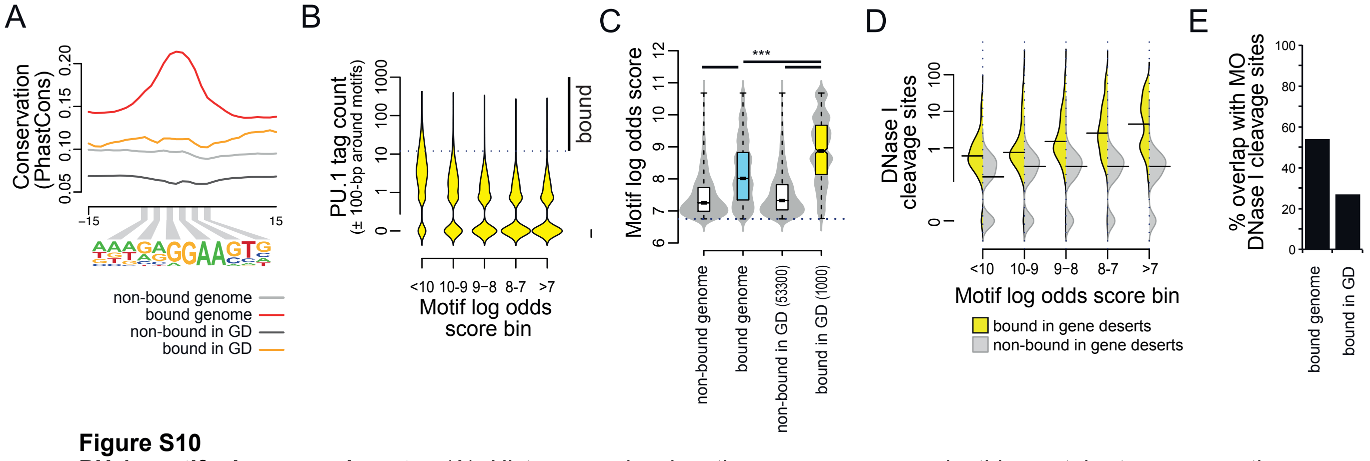

Figure S10

PU.1 motifs in gene deserts. (A) Histogram showing the average per-nucleotide vertebrate conservation (PhastCons) surrounding PU.1 motifs in gene deserts or across the entire genome. (B) Bean plots showing the distribution of normalized PU.1 ChIP-seq tag counts across PU.1 motifs in motif score classes. (C) Combined bean & box plot showing the distribution of motif log-odds scores of non-bound or bound PU.1 motifs across the whole genome or in gene deserts only. Solid bars of boxes display the interquartile ranges (25–75%) with an intersection as the median; whiskers, max/min values. Significantly different motif score distributions in pairwise comparisons are indicated (*** $P < 0.001$, Mann-Whitney U test, two-sided). (D) Bean plots showing the distribution of DNase I cleavage frequency around PU.1 bound (yellow filling) and non-bound motifs (gray filling) in gene deserts depending on motif score classes. DNase cleavage sites (at nucleotide resolution) were counted in a 200-bp window around each motif. Horizontal bars mark the median of each distribution. DNase I cleavage data (representing four independent donors) were originally generated by the ENCODE or the Roadmap Epigenomics projects (all high-throughput sequencing data sets used in this study are listed in Table S1). (E) Frequency of bound PU.1 motifs overlapping DNase I accessible sites in MO.

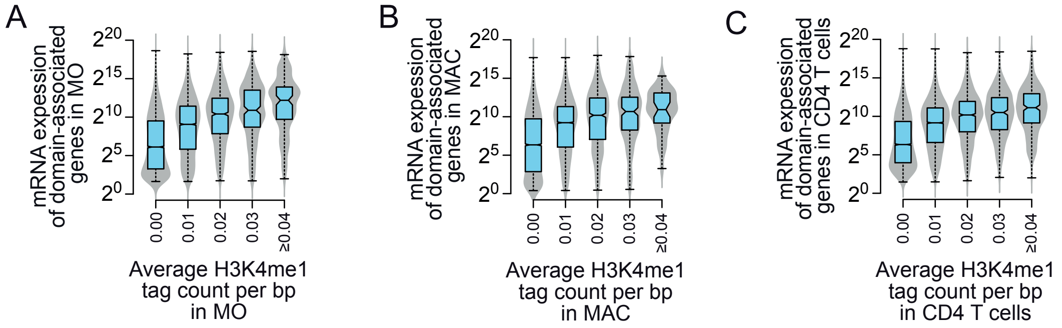

Figure S11

Distributions of gene expression levels in CTCF-flanked domains contingent of their H3K4me1 level. Combined bean & box plot showing the distribution of mRNA expression levels (based on published microarray data) of domain-associated genes in MO (A), MAC (B) and CD4 T cells (C). Solid bars of boxes display the interquartile ranges (25–75%) with an intersection as the median; whiskers, max/min values.

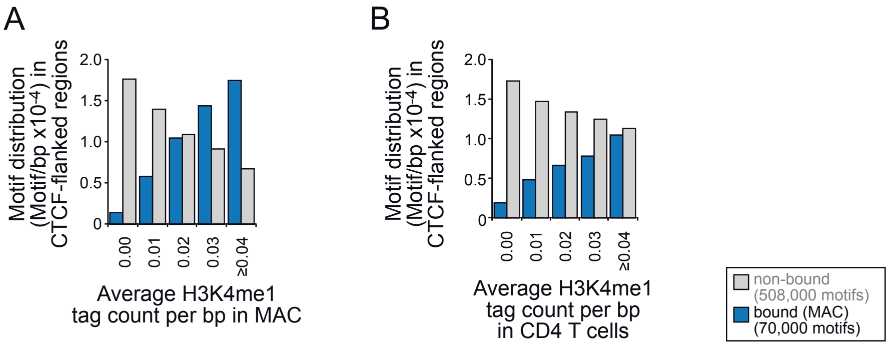

Figure S12

Motif distribution in CTCF-flanked domains contingent of their H3K4me1 level. Bar charts of motif frequencies across domain activity bins for MAC (A) and T cells (B). CTCF-flanked regions were binned according to their H3K4me1 levels in MAC or T cells. In both cases, binding events in MAC were counted (since T cells don't express PU.1).

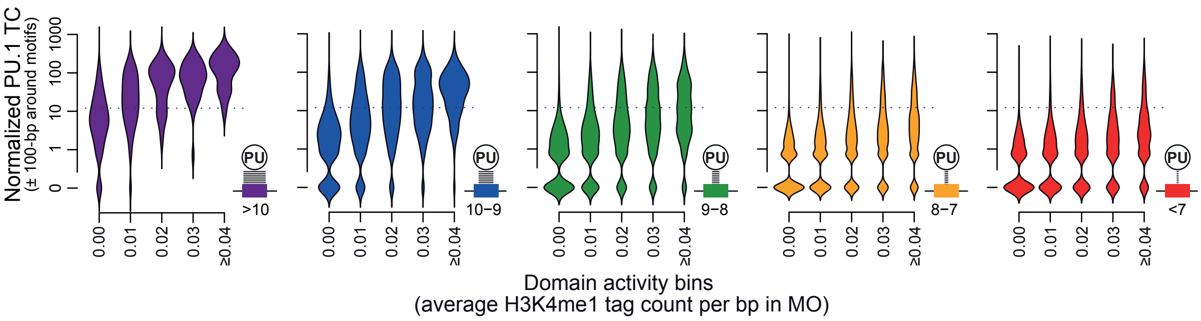

Figure S13

Distribution of motif-associated PU.1 tag counts in CTCF-flanked domains contingent of their H3K4me1 level. The PU.1 PWM was mapped across the masked human genome and all recognized sites were binned contingent on their motif log-odds scores and their location in CTCF-flanked domains. Bean plots show the distribution of PU.1 ChIP-seq tag counts (TC) associated with motifs. High score motifs show the highest PU.1 tag counts and are almost always bound in domains with high activity. The dotted line represents a TC of 12, which was used to define binding events.

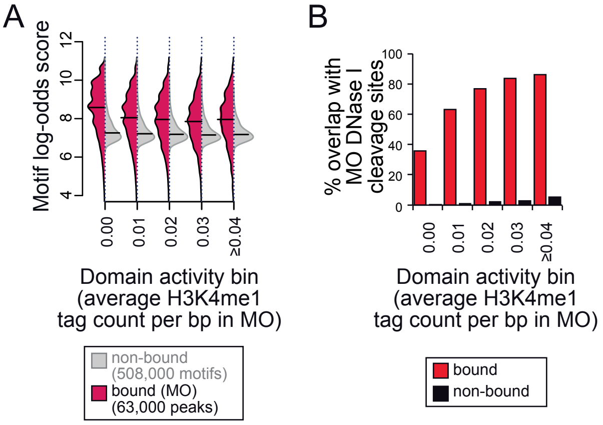

Figure S14

Motif score distribution and DNAse I accessibility in CTCF-flanked domains contingent of their H3K4me1 level. (A) Bean plots showing the distribution of motif log-odds scores for MO PU.1 bound (red filling) and non-bound motifs (gray filling) in CTCF-flanked regions depending on domain activity. Horizontal bars represent medians of motif score distributions. (B) Frequency of bound and non-bound PU.1 motifs overlapping DNase I accessible sites in MO as a function of domain activity.

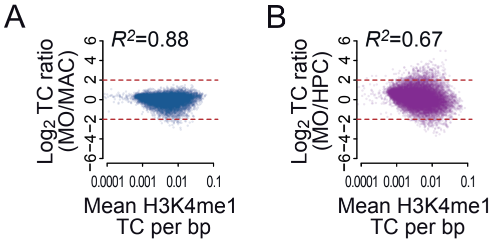

Figure S15

Cell type-specific domain activities in MO, MAC and HPC. Tag count per bp ratios for MO vs. HPC (A) or MO vs. MAC (B) are plotted against average tag counts for CTCF-flanked domains (MvA-plots). The correlation coefficients for direct comparisons of log-transformed tag counts (TC) per bp are given above each diagram.

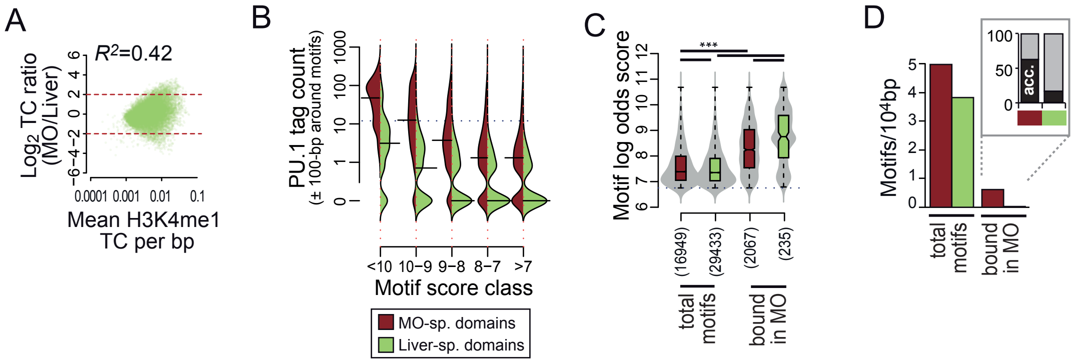

Figure S16

Motif analyses in domains with differential activity between MO and liver. (A) Tag count per bp ratios for MO vs. liver are plotted against average tag counts for CTCF-flanked domains (MvA-plot). The correlation coefficient for the direct comparison of tag counts (TC) per bp are given above the diagram. (B) Distribution of normalized PU.1 ChIP-seq tag counts around motifs contingent on motif score classes in domains showing cell type-specific activity. The horizontal bar indicates the median of each distribution. The dotted line indicates the tag threshold for peaks considered bound. (C) Combined bean & box plot showing the distribution of motif log-odds scores for all (total) motifs or motifs bound in MO in domains showing cell type-specific activity. Solid bars of boxes display the interquartile ranges (25–75%) with an intersection as the median; whiskers represent min/max. Coloring indicates the type of domains tested. Significantly different motif score distributions in pair-wise comparisons are indicated (*** P < 0.001, Mann-Whitney U test, two-sided). (D) Bar chart of total and bound motif frequencies in OB- or MO-specific domains. The additional boxed chart shows frequencies of bound motifs overlapping with DNase I accessible sites in MO.

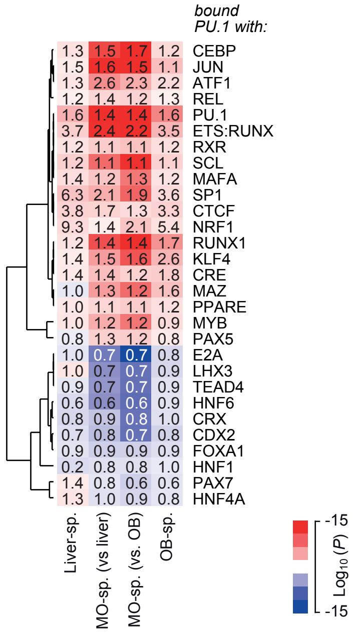

Figure S17

Enrichment of PU.1 co-associated transcription factor consensus sites in domains showing cell type-specific activity. Hierarchical clustering (Pearson correlation uncentered, average linkage) of enrichment values for co-association of the indicated PU.1-bound consensus motifs (within +/- 100-bp) in liver- or osteoblast (OB)-specific domains and MO-specific domains (compared to either liver or OB). P values for motif co-enrichment were calculated using the hypergeometric test relative to the distribution in the total repeat-masked set. Data are presented as a heatmap where red (blue) coloring indicates a significant enrichment (depletion) of motif co-occurrence. Numbers in boxes represent corresponding relative changes in motif co-enrichment.

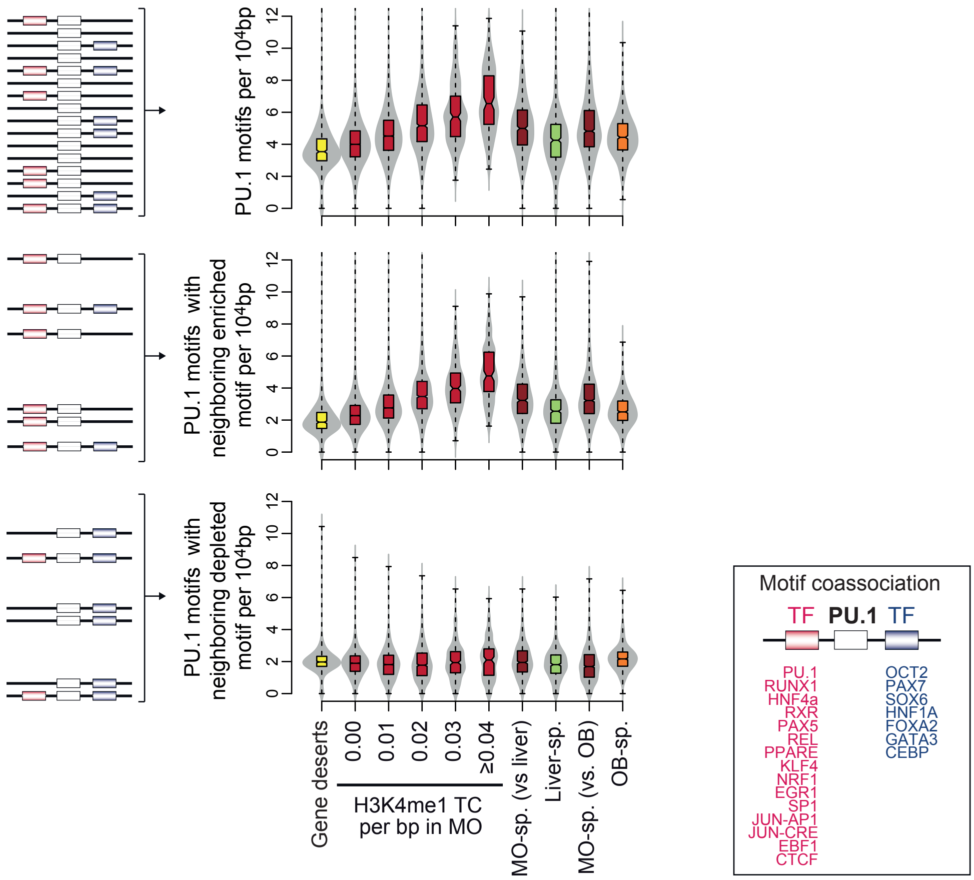

Figure S18

Distribution of PU.1 motifs across domain categories. PU.1 motifs were collected that had at least one of the indicated enriched (red coloring) or depleted motif (blue coloring) in close neighborhood (+/- 100-bp). The combined bean bean & box plots indicate the frequency distributions (motifs/10-kb) of all PU.1 motifs (top panel) or PU.1 motifs that are associated with at least one of the enriched (middle panel) or depleted motifs (bottom panel) in the indicated domain categories. Gene deserts, inactive domains (no H3K4me1 in MO) and domains with specific activity in unrelated cell types (Liver- and OB-specific) are generally characterized by lower densities of PU.1 motifs and fewer PU.1 motifs with coassociated, enriched transcription factor motifs.
